# Cytomolecular Analysis of Ribosomal DNA Evolution in a Natural Allotetraploid *Brachypodium hybridum* and Its Putative Ancestors—Dissecting Complex Repetitive Structure of Intergenic Spacers

**DOI:** 10.3389/fpls.2016.01499

**Published:** 2016-10-14

**Authors:** Natalia Borowska-Zuchowska, Miroslaw Kwasniewski, Robert Hasterok

**Affiliations:** ^1^Department of Plant Anatomy and Cytology, Faculty of Biology and Environmental Protection, University of Silesia in KatowiceKatowice, Poland; ^2^Department of Genetics, Faculty of Biology and Environmental Protection, University of Silesia in KatowiceKatowice, Poland

**Keywords:** 35S rDNA, NOR, allopolyploidy, *Brachypodium hybridum*, intergenic spacer, nucleolar dominance, nucleolus, grasses

## Abstract

Nucleolar dominance is an epigenetic phenomenon associated with nuclear 35S rRNA genes and consists in selective suppression of gene loci inherited from one of the progenitors in the allopolyploid. Our understanding of the exact mechanisms that determine this process is still fragmentary, especially in case of the grass species. This study aimed to shed some light on the molecular basis of this genome-specific inactivation of 35S rDNA loci in an allotetraploid *Brachypodium hybridum* (2*n* = 30), which arose from the interspecific hybridization between two diploid ancestors that were very similar to modern *B. distachyon* (2*n* = 10) and *B. stacei* (2*n* = 20). Using fluorescence *in situ* hybridization with 25S rDNA and chromosome-specific BAC clones as probes we revealed that the nucleolar dominance is present not only in meristematic root-tip cells but also in differentiated cell fraction of *B. hybridum*. Additionally, the intergenic spacers (IGSs) from both of the putative ancestors and the allotetraploid were sequenced and analyzed. The presumptive transcription initiation sites, spacer promoters and repeated elements were identified within the IGSs. Two different length variants, 2.3 and 3.5 kb, of IGSs were identified in *B. distachyon* and *B. stacei*, respectively, however only the IGS that had originated from *B. distachyon*-like ancestor was present in the allotetraploid. The amplification pattern of *B. hybridum* IGSs suggests that some genetic changes occurred in inactive *B. stacei-*like rDNA loci during the evolution of the allotetraploid. We hypothesize that their preferential silencing is an effect of structural changes in the sequence rather than just the result of the sole inactivation at the epigenetic level.

## Introduction

Allopolyploidy has long been recognized as one of the most prominent mechanisms of angiosperm evolution and is often attributed to the increased genetic diversity which may be manifested in the novel traits of a polyploid organism that are not present in its diploid progenitors (Soltis et al., 2015). Although young allopolyploids can evolve into successful species, in the short term the combination of two or more distinct genomes in one nucleus is often associated with various problems, including intergenomic exchanges, sequence loss, transposon proliferation and meiotic irregularities (Parisod et al., 2010; Grandont et al., 2013). The process of allopolyploid stabilization, which is called diploidization, involves changes at both the genetic and epigenetic levels that lead to a diploid-like meiotic behavior and the production of functional gametes as well as a reduced gene expression level in the allopolyploid to a level comparable to its diploid progenitors (Ma and Gustafson, 2005). Several evolutionary scenarios are possible for the homoeologous genes in allopolyploids. It was shown that both of the homoeologues that have been inherited from the two ancestors can be maintained and remain functional in the allopolyploid. It is also possible that one copy accumulates mutations and either gains a new function or becomes repressed. The elimination of one gene variant has also frequently been observed (Tate et al., 2009).

The tandemly repeated 35S rDNA constitutes a molecular and cytogenetic marker of allopolyploidy (Malinska et al., 2010). Each rDNA unit is composed of the three rRNA coding sequences, two internal transcribed spacers (ITSs) and one intergenic spacer (IGS; Volkov et al., 2004; Poczai and Hyvonen, 2010; Shaw, 2013). In contrast to the highly conserved coding sequences, both the IGS and ITSs evolve rather rapidly and as a result their lengths and sequences display considerable variability. The hybrid origin of many plant species that have maintained both parental variants of rDNA long after the formation of allopolyploid has been documented using ITS sequences (O'Kane et al., 1996; Poczai and Hyvonen, 2010). Nevertheless, many allopolyploids suffer from various rDNA rearrangements, including locus and repeat loss as well as interlocus recombination (Wendel et al., 1995; Bao et al., 2010; Kotseruba et al., 2010). The concerted evolution of rDNA loci in the context of allopolyploids may lead to the homogenization of parental units into a single repeat type (Dobesova et al., 2015). Moreover, there is much evidence that the number of rDNA loci is reduced to a diploid-like number during the evolution of an allopolyploid (Kovarik et al., 2008). In many species, such evolutionary processes hamper the effective identification of their hybrid origin, based only on the ITS or IGS sequences (Malinska et al., 2010).

The 35S rRNA genes have also been extensively studied in allopolyploids because of the enigmatic phenomenon of nucleolar dominance (ND; Pikaard, 2000). Its presence, which was originally described as “differential amphiplasty,” was first observed in the interspecific hybrids of *Crepis* in which only the chromosomes that had been inherited from one of the ancestors carried secondary constrictions, while the 35S rRNA gene set from the other ancestor was transcriptionally silenced (Navashin, 1934; Tucker et al., 2010; Ge et al., 2013). To date, ND has been described in the interspecific hybrids of numerous plant genera and in at least one intergeneric hybrid—triticale (Lacadena et al., 1984; Pikaard, 2000). The precise mechanisms that determine which set of rRNA genes is chosen to be silenced in a genetic hybrid remain unclear. It is widely accepted that at least two epigenetic processes, i.e., DNA methylation and histone deacetylation, cooperate in this genome-specific repression of the 35S rRNA gene loci (Chen and Pikaard, 1997; Lawrence et al., 2004; Probst et al., 2004). To discover the exact molecular basis that is responsible for ND, reverse genetics approaches have been undertaken. As a result, various specific chromatin modifiers that play crucial roles in ND were identified, including HDA6 histone deacetylase, DRM2 cytosine *de novo* methyltransferase and two methyl cytosine-binding proteins—MBD6 and MBD10 (Earley et al., 2006; Preuss et al., 2008). Although, recent studies confirmed the epigenetic nature of this phenomenon, the fundamental question of how one set of 35S rRNA genes can be predestined for transcriptional silencing still remains unanswered (Tucker et al., 2010). Moreover, most of the data that is linked with the ND comes from experiments on the dicot plants of the Brassicaceae family, especially the allotetraploid *Arabidopsis suecica*. Despite the studies on cereal hybrids which employed classical cytogenetic methods (Lacadena et al., 1984; Vieira et al., 1990; Neves et al., 1995), little is known about the details of the preferential silencing of rRNA genes in the economically important Poaceae family. It is then important to verify whether the molecular mechanisms of nucleolar dominance in grasses are the same as those in the more extensively studied dicotyledonous allopolyploids.

In this study, complex molecular and cytogenetic approaches were used in order to shed some light on the ND phenomenon in the natural grass allotetraploid, *Brachypodium hybridum* (2*n* = 30). We present the 35S rRNA gene IGS sequence structure of this allotetraploid and its putative ancestors, a model grass *B. distachyon* (2*n* = 10) and *B. stacei* (2*n* = 20). Moreover, the physical localization of the *B. stacei*-like IGS in both metaphase chromosomes and the interphase nuclei that had been isolated from the roots of the allotetraploid is shown. The occurrence of genome-specific rRNA gene silencing in the root apical meristem cells of *B. hybridum*, which was briefly reported by our group (Idziak and Hasterok, 2008), was not only confirmed and further analyzed but was also demonstrated to occur in the differentiated cell fraction in the roots of this allotetraploid.

## Materials and methods

### Plant material and DNA extraction

Plants used in this study were as follows: diploid *B. distachyon* (2*n* = 10) reference genotype Bd21, diploid *B. stacei* (2*n* = 20) genotype ABR114 and three genotypes (ABR113, ABR107 and ABR117) of allotetraploid *B. hybridum* (2*n* = 30). All ABR genotypes were obtained from the collection of the Institute of Biological, Environmental and Rural Sciences (Aberystwyth University, UK), while Bd21 line was received from US Department of Agriculture—National Plant Germplasm System. All plants were grown at 22°C with a 16 h photoperiod in a greenhouse. Total genomic DNA was isolated from young leaves of 1-month-old plants. The tissue was ground in liquid nitrogen and the DNA was extracted using a DNeasy Plant Mini kit (Qiagen) according to the manufacturer's protocol. The quantity and purity of the isolated DNA was determined using a NanoDrop spectrophotometer and was additionally verified using 1% agarose gel electrophoresis.

### PCR amplification, cloning, and sequencing of IGSs

The PCR primers that were used to amplify the IGSs were designed to match the conserved regions of the 18S and 25S rRNA genes (Chang et al., 2010). The IGS was amplified from the genomic DNA of both ancestral species and *B. hybridum* ABR113. Each 20 μL reaction mixture contained 25 ng of total genomic DNA, 200 μM of each dNTP, 0.5 μM of each primer, 10 × reaction buffer with 2 mM MgCl_2_ and 1.5U FastStart Taq DNA polymerase (Roche). The IGS amplification was carried out with an initial denaturation at 98°C for 1 min, followed by 35 cycles of amplification with denaturation at 98°C for 30 s, annealing of primers at 58°C for 15 s and DNA elongation at 72°C for 1.5 min. PCR products were separated by electrophoresis in 1% agarose gel and the selected IGS amplicons were cloned into a pGEM-T Easy Vector System II (Promega). The plasmid DNA from a single recombinant clone for *B. stacei* and *B. hybridum* was isolated using a Qiaprep Spin Miniprep kit (Qiagen). The inserts were sequenced using the Sanger method and the primer-walking strategy (Supplementary Table 1). Since the high repeat content in the intergenic spacer of *B. stacei* hampered its effective sequencing by primer-walking, the *B. stacei* IGS was sequenced using the GS Junior sequencing system (Roche 454).

*B. distachyon* IGS was identified in the entire genome sequence of Bd21 (http://brachypodium.org) with the *B. hybridum* IGS used as a query. Five primer pairs for PCR reactions (Supplementary Table 2) were designed to amplify the fragments of *B. distachyon* IGS and then to resequence the whole intergenic spacer. Each 25 μL PCR mixture contained 20–30 ng of the total genomic DNA of Bd21, 100 μM of each dNTP, 0.4 μM of each primer, 1.5 mM MgCl_2_, a 10 × reaction buffer and 1U Taq DNA polymerase (Promega). The amplification was done with an initial denaturation at 94°C for 1 min, followed by 35 cycles of amplification with denaturation at 94°C for 40 s, primer annealing at 52°C for 40 s and synthesis at 72°C for 80 s. All of the products were sequenced using the Sanger method and assembled into a contig using Geneious software. The IGS sequences are deposited in GenBank under the following accession numbers: KX263276, KX263277, and KX263278.

The dot matrix analyses were performed using the Geneious software. Tandem repeats within all of the studied IGSs were identified using the Tandem Repeat Finder (Benson, 1999). The IGS sequences were aligned using the ClustalW2 program.

### Root meristem preparation

Mitotic chromosome preparations were made according to a previously described procedure (Jenkins and Hasterok, 2007). In brief, the seeds were grown on filter paper moistened with tap water for 72 h at room temperature in the dark. Seedlings with two to three-cm-long roots were treated in ice-cold water for 24 h, fixed in a 3:1 (v/v) methanol:glacial acetic acid at 4°C overnight and stored at −20°C until use. After washing in a 0.01 mmol/L citric acid-sodium citrate buffer (pH 4.8), the roots were digested enzymatically for 1.5 h at 37°C in a mixture of 20% (v/v) pectinase (Sigma-Aldrich) and 2% (w/v) cellulase “Onozuka R-10” (Serva). After digestion, the meristems were dissected from the root tips and squashed in 45% acetic acid. After freezing on dry ice, the cover slips were removed and the preparations were air dried.

### Nuclei isolation

Interphase nuclei from 2-cm-long roots were isolated according to the method described by Lysak et al. (2006). At least 40 seedlings were fixed in 4% formaldehyde in 1 × PBS (pH 7.3) at 4°C for 30 min. After fixation, the seedlings were washed twice in an ice-cold 1 × PBS buffer for 5 min. The roots were separated and washed in a Tris buffer at 4°C for 20 min and chopped with a razor blade in a 400 μL LB-01 buffer on ice in Petri dish. The suspension was filtered through nylon mesh with 30 μm pores and 20–30 μL of nuclei suspension was dropped on ice-cold microscopic slides and air-dried.

### DNA probes and fluorescence *In situ* hybridization

**Figure 1:**
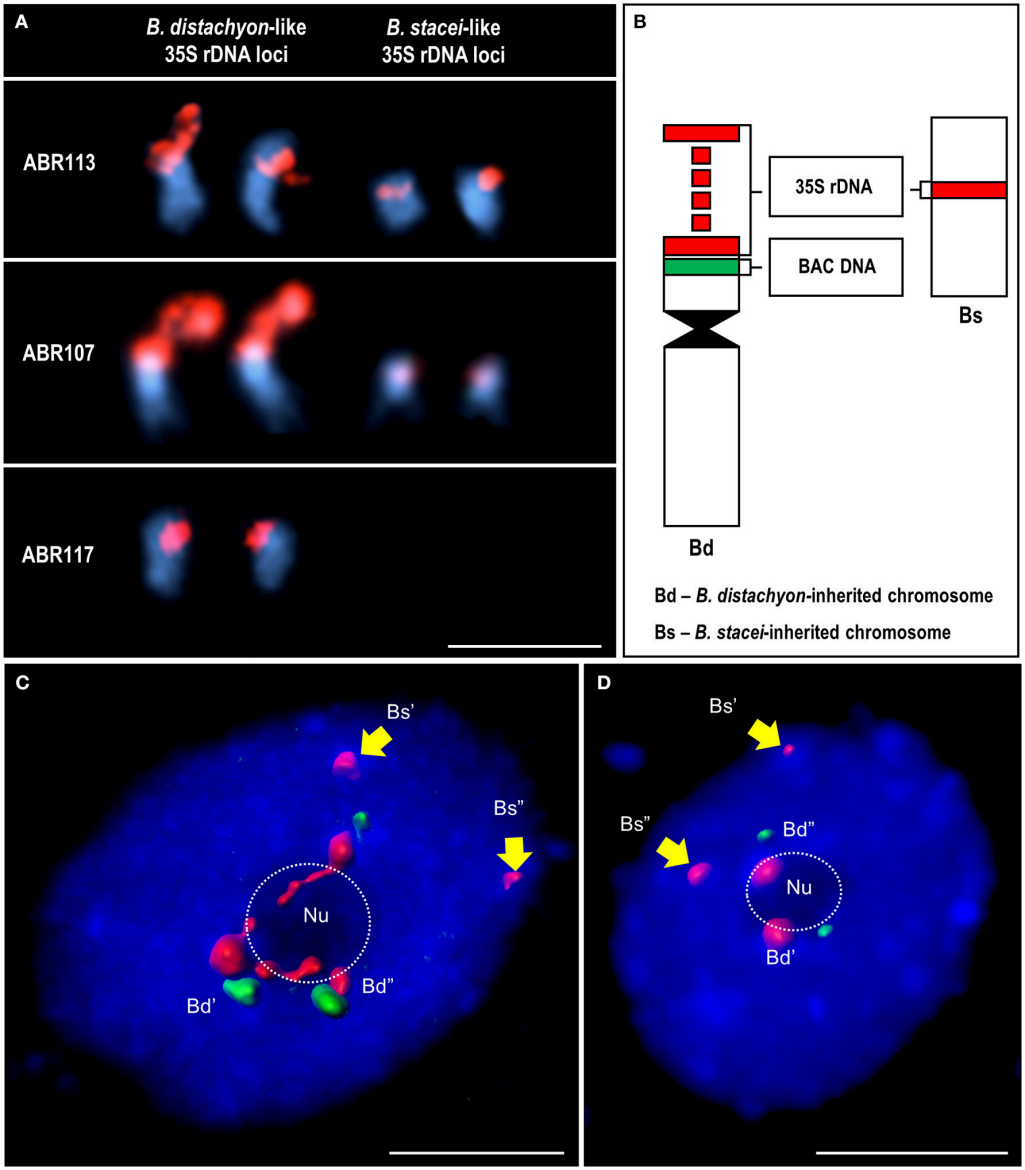
**The localization of 35S rDNA loci inherited from both ancestral species in the somatic metaphase chromosomes and interphase nuclei of ***B. hybridum.*** (A)** Chromosomes carrying the 35S rDNA loci in three *B. hybridum* genotypes: ABR113, ABR107 and ABR117, subjected to FISH with 25S rDNA probe (red). **(B)** Schematic representation of the 35S rDNA-bearing chromosomes of *B. hybridum*. The 35S rDNA loci are red while the position of *B. distachyon*-specific BAC clones (a0019O20 or a0009O09) green. **(C,D)** Interphase nuclei after FISH with 25S rDNA (red) and a0019O20 **(C)** or a0009O09 **(D)** BAC probes. Hybridization signals were modeled using Imaris (Bitplane) software. The position of the nucleoli (Nu) is indicated by a dotted line. Chromatin stained with DAPI (blue). Bars: 5 μm.

A 2.3 kb fragment of the 25S rDNA coding sequence of *Arabidopsis thaliana*, which had been labeled with tetramethyl-rhodamine-5-dUTP (Roche) using nick translation, was used to localize the 35S rRNA gene loci in both the chromosomes and interphase nuclei of the studied species. An amplified IGS sequence from *B. stacei* was labeled with digoxigenine-11-dUTP (Roche) and used as the second probe. Additionally, in order to discriminate in *B. hybridum* interphase nuclei the 35S rRNA gene loci that had been inherited from both ancestors, a 25S rDNA sequence labeled with tetramethyl-rhodamine-5-dUTP and *B. distachyon*-specific low-repeat BAC clones (a0019O20 or a0009O09; Febrer et al., 2010) labeled with digoxigenine-11-dUTP were used. Their localization in the *B. hybridum* chromosomes that bear the 35S rDNA loci is shown in Figure 1B.

The fluorescence *in situ* hybridization (FISH) procedure was adopted from Idziak et al. (2011). Two FISH variants were performed at different stringencies, which reflect the percentage of nucleotide identity between a probe and a target:

i. a reaction with either the BAC clones and 25S rDNA as probes, in which the stringency amounted to 79% or Bs IGS (60.5% GC content) and 25S rDNA with a 70% stringency;
ii. a reaction with the Bs IGS as a probe and more restrictive conditions (87% stringency).

The 25S rDNA and IGS/BAC DNA probes were pooled, precipitated and dissolved in a hybridization mixture that consisted of 50% deionized formamide, 10% dextran sulfate and a 2 × saline sodium citrate (SSC) buffer. In the kinetically more restrictive FISH experiment, the precipitated Bs IGS probe was dissolved in the following hybridization mixture: 60% deionized formamide, 10% dextran sulfate and a 0.5 × saline sodium citrate (SSC) buffer. The mixture was predenatured at 75°C for 10 min, applied to the slides with the chromosome and isolated nuclei preparations and then denatured together at 75°C for 4.5 min. Hybridization was performed overnight at 37°C in a humid chamber. Post-hybridization washes were performed in 10% deionized formamide in 0.1 × SSC for 10 min at 42°C (the equivalent of 79% stringency). In the kinetically more restrictive FISH variant with the Bs IGS probe, 30% deionized formamide in 0.1 × SSC was used at this stage (the equivalent of 86% stringency). The chromosomes and nuclei were counterstained with 2.5 mg/ml 4′,6-diamidino-2-phenylindole (DAPI, Serva) in Vectashield (Vector Laboratories). Photomicrographs were taken using either an AxioCam HRm monochromatic camera attached to a wide-field AxioImager.Z2 epifluorescence microscope (Zeiss) or an Olympus FV-1000 confocal microscope and then processed using MBF ImageJ (NIH, US).

## Results

### Localization of the 35S rDNA loci in the *B. hybridum* metaphase spreads and interphase nuclei

The number and position of the 35S rRNA gene loci were verified by *in situ* hybridization with 25S rDNA in the mitotic chromosome complements of three different genotypes of *B. hybridum* (ABR113, ABR107, and ABR117). It was confirmed that the genotype ABR113 had two chromosome pairs that bear 35S rDNA loci that had been inherited from both ancestral species (Figure 1A). We demonstrated that the genotype ABR107 also had the sum of the 35S rDNA loci that had been expected from the numbers present in the ancestors (Figure 1A); however, the 25S rDNA FISH signals in the *B. stacei-*inherited chromosome pair were significantly smaller than in ABR113. As was shown by Hasterok et al. (2004), a reduction in the number of the 35S rDNA loci occurred in ABR117 (Figure 1A). Only the terminally located 35S rRNA gene loci in the *B. distachyon*-like chromosomes were present in this genotype.

The location of 35S rDNA in the interphase cell spreads of *B. hybridum* ABR113 was determined by FISH with 25S rDNA and chromosome-specific BACs, which preferentially hybridized to the short arm of the NOR-bearing chromosome Bd5 of *B. distachyon* (Figure 1B). The BAC clones used in the study selectively marked only *B. distachyon*-like 35S rDNA loci in both the mitotic metaphase chromosomes and the interphase nuclei that had been isolated from *B. hybridum* roots (Figures 1C,D), thus providing valuable markers that enabled the intergenomic distinction of these loci in the allotetraploid. Noticeably, in all of the studied nuclei, the 25S rDNA loci that had been inherited from *B. stacei* were distributed in the DAPI-positive chromocenters at the nuclear periphery (Supplementary Figure 1). Moreover, these loci were unable to form nucleolus/nucleoli, which was indirect evidence of their transcriptional repression. In contrast, the hybridization signals corresponding to the 25S rDNA loci that had been inherited from the second ancestor tended to be located either within the nucleolus and adjacent to the nucleolus in the chromocenters (Figure 1C; Supplementary Figure 1) or were present only at the nucleolar periphery in the chromocenters (Figure 1D; Supplementary Figure 2). The signals observed within the nucleolus appeared to be more diffused compared to the signals that were present in the chromocenters that adjoined the nucleolus.

### Amplification and sequencing of the IGSs

**Figure 2:**
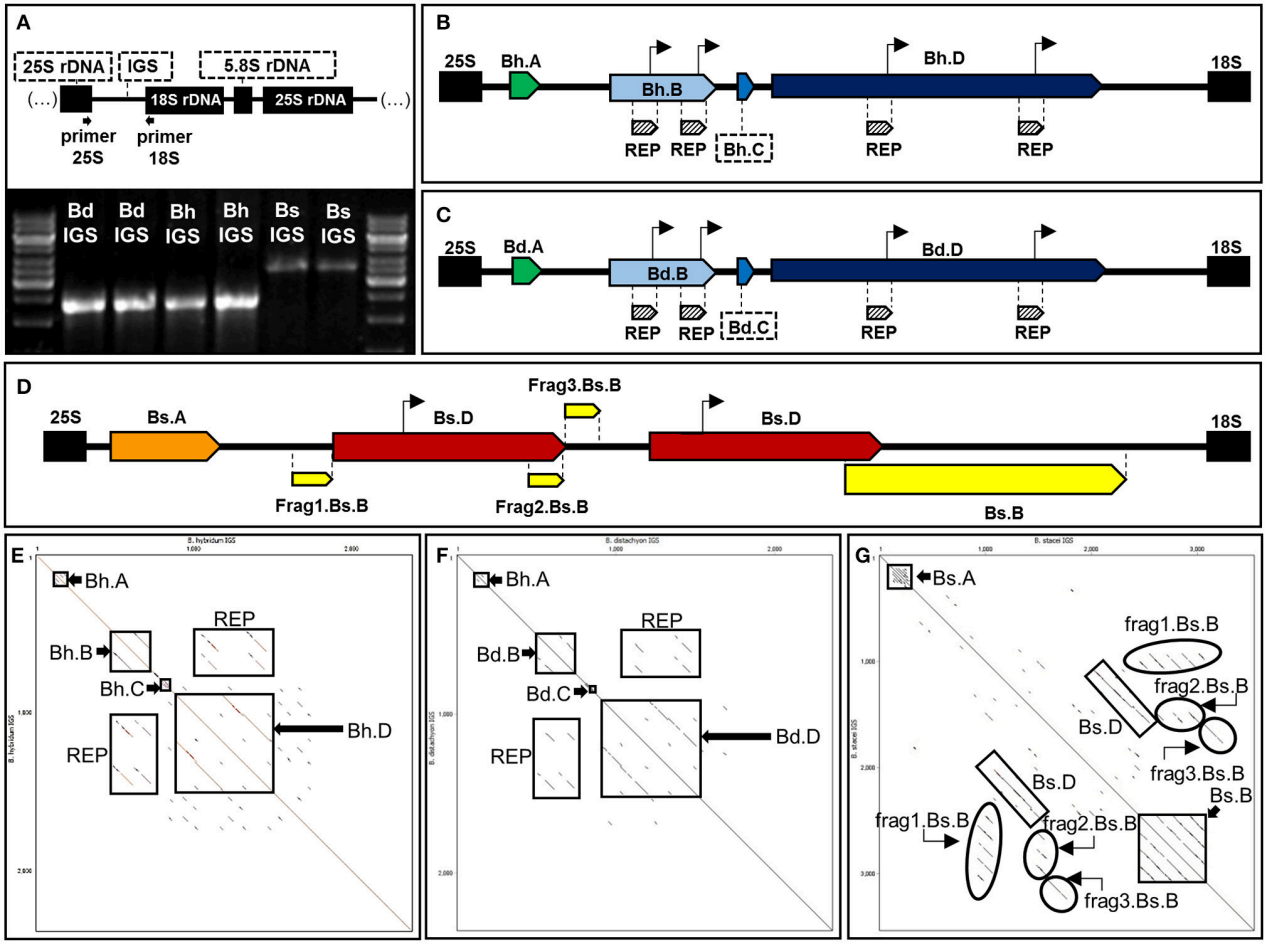
**Structural organization of 35S rDNA intergenic spacers of ***B. hybridum*** (Bh IGS) and the modern relatives of ancestral species ***B. distachyon*** (Bd IGS) and ***B. stacei*** (Bs IGS)**. **(A)** PCR profiles of the intergenic spacers. The position of primers that were used for PCR is indicated below the diagram of the 18S-5.8S-25S rDNA unit. Bright bands in the marker line reflect 6 kb (upper band) and 3 kb (lower band). **(B,C)** Schematic representation of Bh IGS **(B)**, Bd IGS **(C)** and Bs IGS **(D)**. Repetitive sequences in each intergenic spacer are denoted as pentagons with different colors. Putative transcription initiation sites (TISs) are marked as arrows over the diagrams. **(E–G)** Dot matrix plots of the intergenic spacers. Self-comparisons of Bh IGS **(E)**, Bd IGS **(F)**, and Bs IGS **(G)**. Position of the repetitive sequences is indicated by rectangles.

PCR with the primers anchored in the highly conserved regions of 25S and 18S rDNA (Supplementary Table 1) was used to amplify the IGSs from *B. hybridum* and its putative ancestors. The amplification with specific, hot-start FastStart DNA polymerase (Roche) produced a clearly defined, single product for all of the species (Figure 2A). In the case of intergenic spacers of *B. distachyon* (Bd IGS) and *B. hybridum* (Bh IGS), PCR products were observed at approximately 2.5 kb while the presence of an ~3.5 kb-long product was identified for *B. stacei* (Bs IGS). Surprisingly, the Bs IGS length variant was not amplified from the gDNA of the allotetraploid (Figure 2A). The lack of Bs IGS strongly suggests that some changes took place at the genetic level in the transcriptionally inactive *B. stacei*-like 35S rDNA loci during the evolution of *B. hybridum*. Such changes most probably involved the primer binding sites.

**Figure 3:**
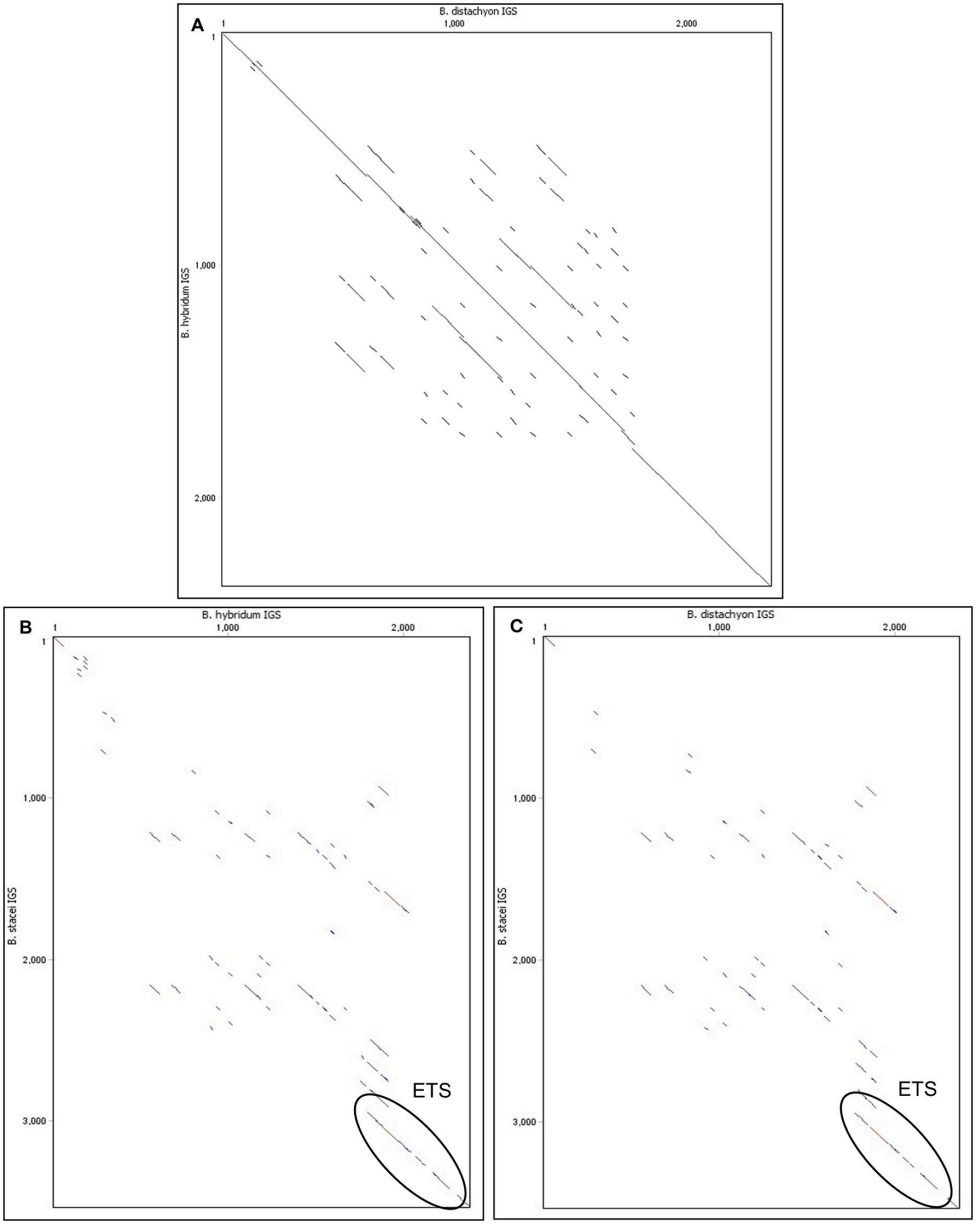
**Comparison of the 35S rRNA gene intergenic spacers between ***Brachypodium*** species on dot matrix plots. (A)** Comparison of Bd IGS vs. Bh IGS **(B)** Comparison of Bh IGS vs. Bs IGS. **(C)** Comparison of Bd IGS vs. Bs IGS.

Intergenic spacers from both modern relatives of the ancestral species (Bd and Bs IGSs) as well as the shorter length variant of the IGS from *B. hybridum* were sequenced and analyzed. The length of the Bh, Bd, and Bs IGSs was 2305, 2282, and 3456 bp, respectively. All of the studied IGSs were characterized by a high guanine-cytosine (GC) content that averaged between 60 and 63%. A sequence comparison between the Bd and Bh IGSs revealed a 95% identity, which further confirmed that the Bh IGS was inherited from *B. distachyon* (Figure 3A). A comparison with an intergenic spacer of *B. distachyon* using the blastn algorithm revealed 41 transitions in the Bh IGS, among which 30 involved changes between pyrimidines and the remaining 11 between purines (Supplementary Figure 3). Moreover, the presence of four transversions, four indels and four gaps that encompassed at least 7 bp was confirmed in the Bh IGS (Supplementary Figure 3).

The intergenic spacer from the second ancestor (Bs IGS) was compared with the Bh IGS (Figure 3B) and the Bd IGS (Figure 3C) on dot matrix plots. In both cases, the only similar region was the 500 bp fragment of the external transcribed spacer (ETS) located at the 3′ end of the IGSs (Figures 3B,C). Blastn analysis of the studied IGSs confirmed that the ETS is conserved among different *Brachypodium* species (Supplementary Tables 3–5). However, this analysis did not reveal any homology to the IGS sequences from other grass representatives that do not belong to the genus *Brachypodium*.

### Structure and functional domains of the IGSs

A 17 bp pyrimidine-rich sequence was identified at the 5′ end of all of the analyzed intergenic spacers. Similar sequences have been denoted in the IGSs of many other plants from different genera, including *Quercus* (Bauer et al., 2009; Inácio et al., 2014), *Solanum* (Borisjuk and Hemleben, 1993), *Arabidopsis* (Gruendler et al., 1991), *Brassica* (Yang et al., 2015), and *Chenopodium* (Maughan et al., 2006), which suggests that they may be involved in the termination of 35S rDNA transcription (Inácio et al., 2014).

**Figure 4:**
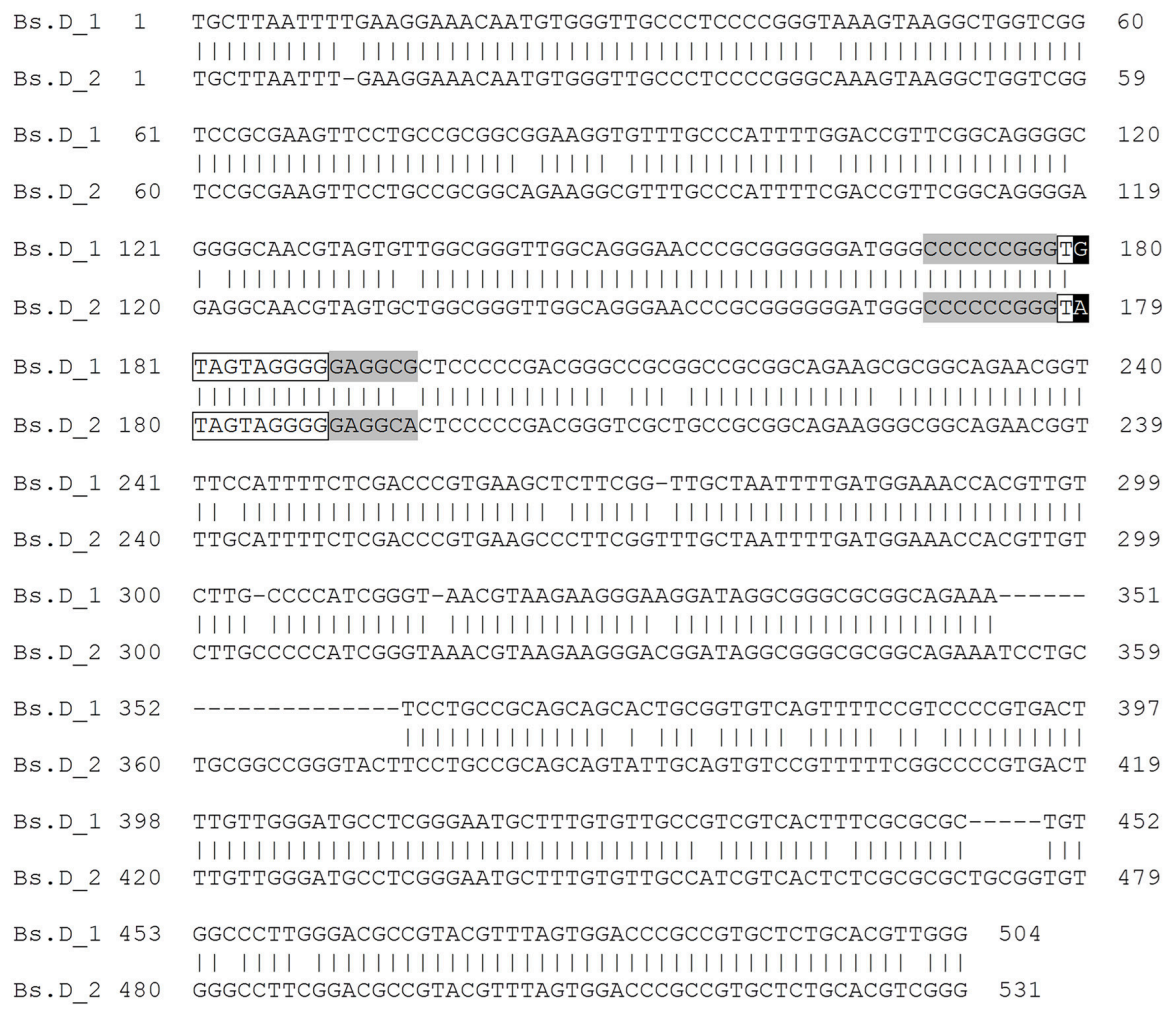
**Blastn alignment of the consecutive motifs of the Bs.D repeat class from ***B. stacei*** IGS**. The TATA sequences are shown in black frames, while TIS flanking regions are highlighted in gray. The substitution within the TATA box sequences is denoted by white print on a black background.

Sequence analysis with both dot matrix plot and Tandem Repeats Finder software revealed that all of the studied IGSs contained repetitive motifs (Figures 2B–G). The detailed description of the identified repeats is shown in Table 1. It is worth noting that some of the repeated sequences contained a TATA box. In the repeats Bh.B and Bd.B for instance, the TATA sequence was identified at the end of each repeated motif (Figures 2B,C). The alignment between the corresponding copies of the Bd.B and Bh.B using ClustalW2 revealed the presence of two substitutions in the first motifs; however, these changes did not involve the TATA sequence (Supplementary Figure 4A). A self-comparison analysis on the dot matrix plots of the Bh and Bd IGSs revealed the presence of additional, long repeat types, which were denoted as Bh.D (Figure 2E) and Bd.D (Figure 2F), respectively. Both identified repeats also contained a TATA box near the 3′ end of each repeated motif. A ClustalW2 alignment of the Bh.D and Bd.D repeats is presented in Supplementary Figure 4B. Several substitutions were detected in the corresponding copies of Bh.D, but none of them involved the TATA sequences. Interestingly, the flanking region of the TATA sequences that were identified in both the Bh and Bd IGSs was conserved and denoted as REPs within the B and D repeat types (Figures 2B,C). In the case of Bs IGS, the TATA box was identified in all of the Bs.D motifs. The TATA box in the second Bs.D copy was identical to the putative transcription initiation sites (TISs) in the Bd and Bh IGSs; however, a single substitution was found in the TATA box from the first copy (Figure 4).

**Table 1:** **Characterization of the repetitive motifs present in the 35S rDNA IGSs of the studied ***Brachypodium*** species**.

**Sequence ID**	**Nucleotide position**	**Motif length [bp]**	**Copy number**	**Interval sequence [bp]**	**Identity between consecutive motifs [%]**	**Presence of TATA box**	**Counterpart in Bh IGS**
**Bh IGS**							
**Bh.A**	120–180	26	2.3	−	78	−	
**Bh.B**	489–720	124	1.9	−	85	+	
**Bh.C**	804–834	8	4.0	−	91	−	
**Bh.D**	887–1479	302	2.0	−	91	+	
**Bd IGS**							
**Bd.A**	120–180	26	2.3	−	73		**Bh.A**
**Bd.B**	489–731	125	1.9	10	85	+	**Bh.B**
**Bd.C**	822–852	8	4.0	−	95	−	**Bh.C**
**Bh.D**	905–1479	303	2.0	−	90	+	**Bh.D**
**Bs IGS**							
**Bs.A**	127–308	26	6.8	−	81	−	−
**Bs.B**	2441–3060	156	3.9	−	86	−	−
**Bs.D**	1071–1574; 2013–2543	531	2.0	439	90	+	−

The colors in the table reflect the colors of pentagrams representing different repetitive elements in Figures 2B–D.

**Figure 5:**
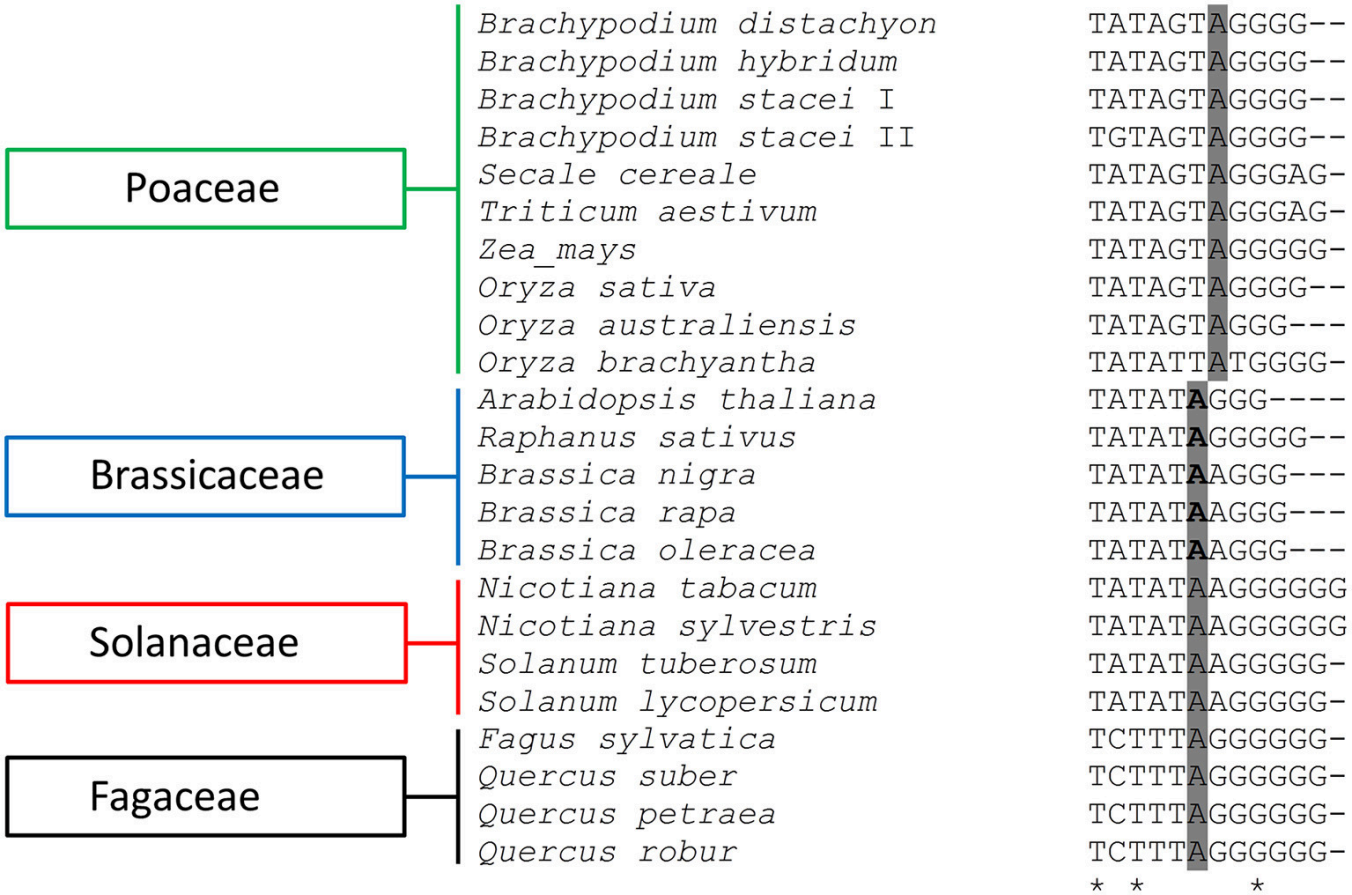
**Comparison of the putative transcription initiation sites (TIS) of different plant species**. The presumptive +1 nucleotide position is indicated in gray, while the experimentally confirmed +1 position are underlined in bold. The asterisks denote positions at which all query sequences have the same residue.

The alignment of the putative TIS that was present in the IGSs of the *Brachypodium* species with the available rRNA gene promoter sequences of the other plants revealed a high sequence conservation around the transcription initiation sites, not only between closely related species but even between monocots and dicots as well (Figure 5). The putative TIS from the *Brachypodium* IGSs showed the highest identity with its counterparts from other Poaceae representatives such as wheat, rye and rice (Figure 5).

### Chromosomal and nuclear distribution of the IGSs

**Figure 6:**
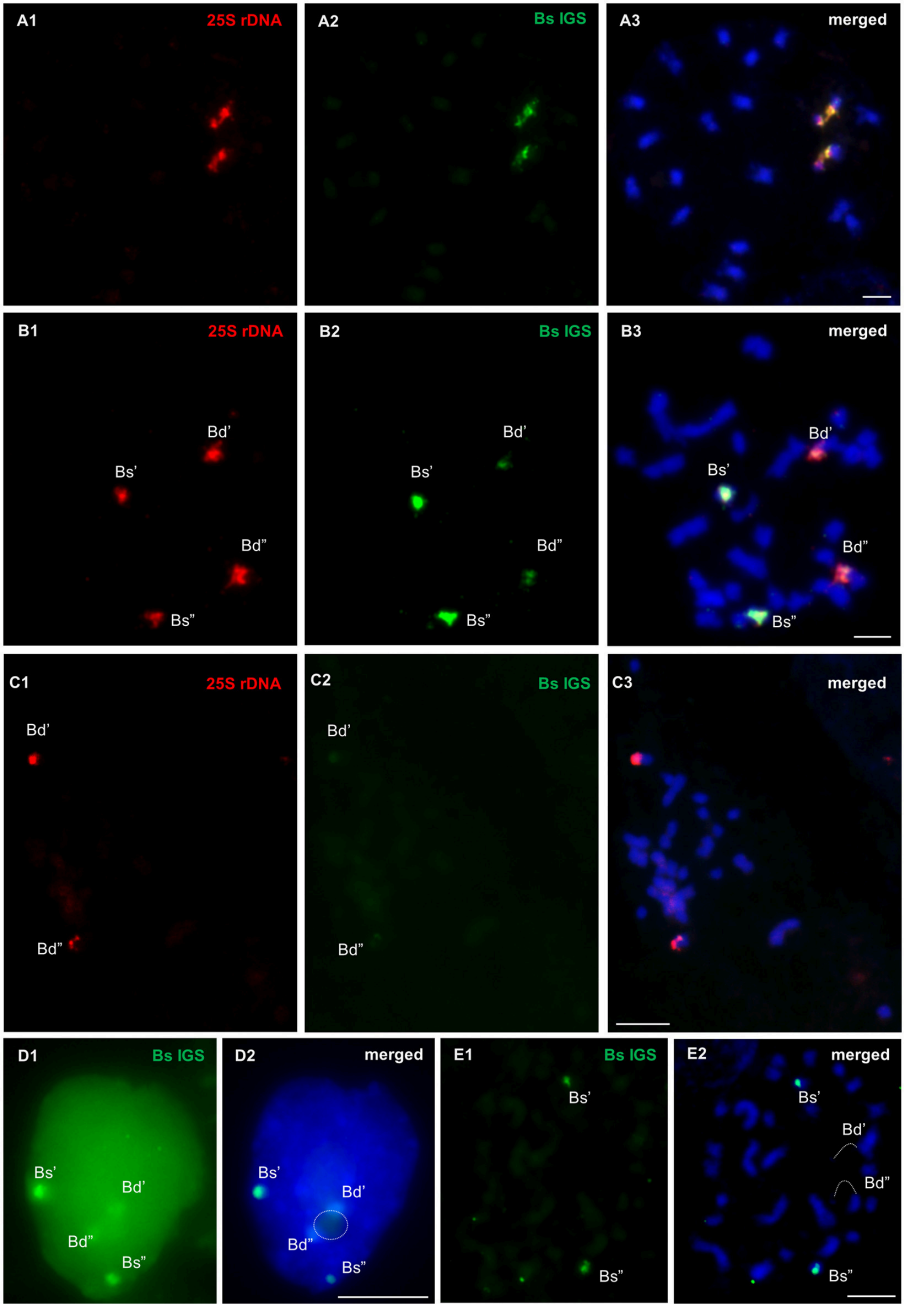
**FISH mapping of ***B. stacei*** IGS (green) and 25S rDNA (red) probes in the metaphase chromosome complements of ***B. stacei*** (A1–A3) and ***B. hybridum*** ABR113 (B1–B3, E1,E2) and ABR117 (C1–C3) as well as in the interphase nuclei that were isolated from ***B. hybridum*** roots (D1,D2)**. Stringency of the FISH experiments: 70% **(A1–A3, B1–B3, C1–C3, D1,D2)** or 87% **(E1,E2)**. Chromatin stained with DAPI. Bars: 5 μm.

The location of the *B. stacei* intergenic spacer in the metaphase chromosomes and nuclei of two *B. hybridum* genotypes (ABR113 and ABR117) was determined using FISH with 25S rDNA and the Bs IGS as probes. FISH on the mitotic metaphase spreads of *B. distachyon* and *B. stacei* constituted the negative and positive control, respectively. As was expected, two bright signals corresponding with the Bs IGS were present in the *B. stacei* NOR chromosomes (Figures 6A1–A3). In the case of *B. distachyon*, two weak Bs IGS hybridization signals were colocalized with 25S rDNA on chromosome Bd5. The presence of the Bs IGS signals on the *B. distachyon* NOR chromosomes may be attributed to the partial homology of the 3′-ETS sequence, which is present in both the Bd and Bs IGSs. In the metaphase chromosome complement of *B. hybridum* ABR113, four Bs IGS signals of different sizes were detected. A pair of strong hybridization signals, which were colocalized with 25S rDNA, was revealed on the *B. stacei*-like chromosomes, while another and relatively weak signal pair was present on the NOR chromosomes that had been inherited from a *B. distachyon*-like ancestor (Figures 6B1–B3). A similar FISH experiment was performed on the metaphase plates of the genotype ABR117 in which only one chromosome pair, which had been inherited from *B. distachyon*, bore 35S rDNA loci. Two weak hybridization signals, which corresponded with the Bs IGS, were observed in the NOR chromosomes of this genotype (Figures 6C1–C3), thus indicating that the Bs IGS variant is absent in ABR117.

In the interphase nuclei of *B. hybridum* (genotype ABR113), only a faint pair of the Bs IGS signals was found next to the nucleolus. Strong FISH signals of the *B. stacei*-like rDNA loci were located at the nuclear periphery (Figures 6D1,D2 and Supplementary Figure 5). In the FISH experiment with the stringency increased to 87%, only two bright hybridization signals, which corresponded with the Bs IGS, were detected in the *B. stacei*-like 35S rDNA bearing chromosomes (Figures 6E1,E2).

## Discussion

### Nucleolar dominance in *B. hybridum* roots?

It is assumed that *B. hybridum* arose from the interspecific cross between *B. distachyon* and *B. stacei* about 1 Mya (Catalan et al., 2012). In 2008, Idziak and Hasterok revealed the presence of ND in the root apical meristems of six *B. hybridum* genotypes for the first time. In all of the analyzed allotetraploid lines, four 35S rDNA loci were observed: two were distributed terminally in the *B. distachyon*-inherited chromosome pair and two smaller ones, which were located in the proximal parts of the *B. stacei-*like chromosomes. It was confirmed that only the *B. distachyon*-like 35S rDNA loci were transcriptionally active in *B. hybridum*, while the loci that had originated from the second ancestor were consistently suppressed (Idziak and Hasterok, 2008). In the present work, the position of the 35S rDNA loci in the interphase nuclei isolated from *B. hybridum* roots was determined. It was found that only the rDNA loci that had originated from the *B. distachyon*-like genome were associated with the nucleolus, while the loci that had been inherited from another parental species were present at the nuclear periphery and were not able to form a nucleolus. The association of the 18S-5.8S-25S rRNA gene loci with a nucleolus/nucleoli provides indirect evidence of their transcriptional activity and that the position of the nucleolus/nucleoli is determined by the location of competent, transcriptionally active rDNA loci (Shaw, 2013). This correlation, which was initially observed by McClintock (1934), was later confirmed by a number of studies on different plant species such as wheat (Leitch et al., 1992), *A. suecica* (Earley et al., 2006), *Quercus robur*, and triticale (Bockor et al., 2014). Since the distribution of 35S rDNA was also analyzed in the nuclei from differentiated cells in the present study, we can assume that the preferential silencing of the *B. stacei*-like rDNA loci is present in the differentiated cell fraction of *B. hybridum* roots as well.

Interestingly, the FISH signals that corresponded with the *B. distachyon*-inherited rDNA loci were not only situated within the nucleolus, but also in the highly condensed, DAPI-positive chromocenters that adjoined the nucleolus (Figure 1C). Such a distribution of hybridization signals suggests the separation of the transcriptionally active and silenced 35S rRNA gene copies that had originated from a common locus. Although, French et al. (2003) revealed that actively transcribed and silenced rDNA copies in yeast NORs are interspersed with one another, studies on plants proved that active and suppressed rRNA genes can occupy distinct NOR portions (Caperta et al., 2002; Pontvianne et al., 2013). It was found, for instance, that silver-stained, decondensed regions do not encompass the entire NOR in rye chromosomes. The condensed, transcriptionally inactive NOR fractions are located next to the secondary constriction of the chromosome in this species (Caperta et al., 2002). In case of the interphase nuclei, it was shown that a single NOR can be composed of both condensed, silent rRNA genes that are situated externally to the nucleolus as well as actively transcribed rRNA genes that are dispersed within the nucleolus (Pontvianne et al., 2013). The rDNA copies in *A. suecica* and *Q. robur*, which are excluded from nucleolus, are enriched by heterochromatic histone modifications (Lawrence et al., 2004; Earley et al., 2006; Bockor et al., 2014). Both the epigenetic and transcriptional states of particular rDNA copies are reversible and depend on the needs of the cell.

It has been proven that ND may be developmentally regulated. Chen and Pikaard (1997) revealed that the 35S rRNA genes that were not expressed in the vegetative tissues of *Brassica napus* were transcriptionally active in all of the floral organs of this allotetraploid, including the petals and sepals. The transition of inflorescence to the floral meristem led to the transcriptional activation of previously silenced rRNA genes (Chen and Pikaard, 1997). Interestingly, in contrast to *B. hybridum* in which ND is present in the root apical meristems (Idziak and Hasterok, 2008), the absence of this phenomenon in root meristematic cells was observed in *Brassica* allotetraploids (Hasterok and Maluszynska, 2000). The question of whether or not the ND pattern in *B. hybridum* is developmentally regulated is worth consideration, although the presence of this phenomenon in both the meristematic and differentiated cells of *B. hybridum* roots suggests that this genome-specific silencing may not be reversible in this species.

### Genetic changes may be behind genome-specific silencing of the *B. stacei*-inherited rDNA loci in *B. hybridum*

In order to verify whether the preferential silencing of the *B. stacei*-inherited rRNA genes in the studied allopolyploid is connected with some genetic changes within the ribosomal DNA loci, we amplified and further analyzed the 25S-18S rDNA IGSs of *B. hybridum* and its putative ancestors. The complete IGS sequences of *Brachypodium* species and their structural organization were deciphered for the first time.

Interestingly, only one length variant of the IGS, which corresponds with the *B. distachyon*-like rDNA locus, was amplified in *B. hybridum*, thus suggesting that the conserved primer binding sites that are located within the conserved regions of 18S and 25S rRNA genes had mutated. Such an IGS amplification pattern in *B. hybridum* suggests either a homogenization process of rRNA genes in the hybrid or an accumulation of mutations in the *B. stacei*-inherited rDNA, which results in the loss of its function. There is evidence that the homogenization of rDNA units in allopolyploids is not accompanied by nucleolar dominance (Kovarik et al., 2008). Studies on two *Nicotiana* allotetraploids, *N. rustica* and *N. tabacum*, revealed that the rDNA units, which did not undergo gene conversion, were transcriptionally silenced (Dadejová et al., 2007). It is well known that repressed rRNA genes are highly methylated, especially at the promoter regions and are enriched by the histone modifications that are characteristic of heterochromatin, e.g. H3K9me2 (Lawrence et al., 2004). Kovarik et al. (2008) postulated that the silenced rDNA loci that are present in heterochromatin are characterized by a lower susceptibility to the homogenization process. Such loci may have been lost during the evolution of an allopolyploid. Taking into account these findings, the conversion of the *B. stacei*-inherited rDNA units appears to be less likely than an accumulation of mutations in the repressed rDNA loci. We can speculate that the process of the deactivation of the *B. stacei*-inherited rDNA during the evolution of *B. hybridum* may have consisted of several distinct stages. The first one, which is the “nucleolar dominance” stage, is characterized by the reversible, epigenetic silencing of the *B. stacei*-like rDNA after the formation of the allotetraploid. In the next “genetic changes” stage, a gradual accumulation of mutations in the repressed rDNA loci leads to the loss of their function. Finally, at the “elimination” stage, the physical loss of inactive *B. stacei*-inherited rDNA might occur.

This hypothesis is supported by the fact that there is at least one *B. hybridum* genotype (ABR117) that exists in which the diploid-like number of the 35S rDNA loci was revealed (Figure 1A). In 2004, Hasterok et al. showed that the *B. stacei*-inherited 35S rDNA loci were completely undetectable in the chromosomes of this genotype. In the present paper, we also indicated that the Bs IGS variant is absent in ABR117 (Figures 6C1–C3). Interestingly, a significant disproportion in the size and intensity of 25S rDNA FISH signals was observed between *B. distachyon-*like and *B. stacei-*like chromosomes in two other genotypes of *B. hybridum*, ABR100 (Hasterok et al., 2004) and ABR107 (Figure 1A). Moreover, the 25S rDNA hybridization signals in the *B. stacei-*inherited chromosomes in both ABR100 and ABR107 were apparently smaller and weaker compared with the corresponding signals in the chromosomes of the genotype ABR113, which may corroborate the hypothesis of the gradual elimination of the inactive ribosomal DNA loci in *B. hybridum*. The reduction of the rDNA loci to a diploid-like number was also confirmed in the allopolyploid *Nicotiana* species from the sections *Polydicliae* and *Repandae*, which are estimated to be ~1 Myr and 4.5 Myr old, respectively (Clarkson et al., 2005; Kovarik et al., 2008).

### Putative TIS regions in IGSs seem to be evolutionary conserved among grasses

The length of the IGSs of all of the studied *Brachypodium* species averaged between 2.3 and 3.5 kb. The main culprit that is responsible for the length (from 1 to 13 kb) heterogeneity of the IGS is the different copy number of the subrepeats that are present within the spacer (Rogers and Bendich, 1987b; Polanco and De La Vega, 1997; Maughan et al., 2006). For instance, interspecific IGS diversity was found for different representatives of Fabaceae, including *Phaseolus* (Schiebel et al., 1989; Maggini et al., 1992), *Vicia faba* (Yakura et al., 1984; Rogers and Bendich, 1987a; Kato et al., 1990), and *Pisum sativum* (Polans et al., 1986; Piller et al., 1990). In contrast, comparative studies of different *Lens* species revealed that some motifs, which are attributed as functional sequences, are conserved in both the sequence and position context (Fernandez et al., 2000; Fernández et al., 2005). Taking this data into account, only the 500 bp sequence, which represents the ETS region, is shared between Bd and Bs IGSs. When the Bs IGS was used as the FISH probe on either the metaphase chromosomes or interphase nuclei of *B. hybridum*, two additional weak hybridization signals were observed in the *B. distachyon*-inherited NOR chromosomes (Figures 6B1–B3,D1,D2), which most probably indicates the presence of a common ETS. The application of more restrictive stringency of the reaction led to the hybridization of this probe only with the *B. stacei*-inherited chromosomes.

The IGSs that separate adjacent rRNA genes contain both the transcription initiation site (TIS) and termination site (TTS), and therefore they play an important role in the transcriptional regulation of the downstream genes. The TISs in some (though not all) rRNA gene promoters are similar in the sequence context and contain a TATA box (Cordesse et al., 1993; Chang et al., 2010). Comparison of the putative TISs for Pol I of the studied *Brachypodium* species revealed the highest identity with the corresponding regions of other Poaceae representatives. In both the Bd and Bh IGSs, three spacer promoters, which were similar in sequence to the gene promoter, were found. In the corresponding region of *B. stacei*, only one putative spacer promoter was observed. Similar to rice (Cordesse et al., 1993), all of the putative TISs were placed within repetitive sequences in *Brachypodium* species. Spacer promoters that have blocks of tandemly repeated sequences represent putative transcription enhancers (Schlögelhofer et al., 2002; Castiglione et al., 2013). Their role in establishing ND was revealed in *Xenopus* hybrids, in which the 35S rDNA loci that had longer IGS and contained more subrepeats upstream to the TIS, dominate over the loci with a lower number of subrepeats. According to this hypothesis, subrepeats together with spacer promoters act as enhancers that have a different transcription factor binding affinity (Reeder and Roan, 1984; Reeder, 1985; Caudy and Pikaard, 2002). In plants, differences in the type and number of IGS repeats have also been proposed to control the activity of the rRNA genes in the hybrids of *Triticum* and representatives of related genera (Martini et al., 1982; Gustafson et al., 1988; Houchins et al., 1997). However, the preferential suppression of the 35S rRNA genes in allopolyploids does not always correlate with the presence of a lower number of putative enhancers within the IGS of the under-dominant genes, as was shown for *Brassica* allotetraploids (Chen and Pikaard, 1997). Collectively, a comparison of the data obtained for cereal hybrids and Brassicaceae allopolyploids showed that the exact molecular mechanism of ND is still unclear and the role of the IGS repeats should be taken into consideration in the different organisms which exhibit this phenomenon.

Despite the fact that our studies strongly support the hypothesis of the inactivation of *B. stacei*-inherited rRNA gene loci at the genetic level, the involvement of other factors cannot be ruled out. The verification of the presence of ND in the floral organs should raise the question of whether this genome-specific inactivation of ribosomal DNA is a reversible, epigenetically regulated process or not. Moreover, the upcoming data about the whole genome sequence of *B. hybridum* should shed more light on the fine-scale organization of the silenced rDNA loci in this allotetraploid.

## Author contributions

Conceived and designed the study: NB, MK, RH; Performed the experiments: NB, MK; Analyzed the data: NB, MK, RH; Wrote the manuscript: NB, RH.

## Funding

The authors gratefully acknowledge the financial support from the National Science Centre, Poland [grant no. DEC-2011/01/B/NZ3/00177 and DEC-2012/04/A/NZ3/00572].

### Conflict of interest statement

The authors declare that the research was conducted in the absence of any commercial or financial relationships that could be construed as a potential conflict of interest.

## Supplementary material

Supplementary Figure 1 **Confocal sections through the interphase nucleus of ***B. hybridum*** that is presented in Figure 1C. (A1–A6)** DAPI counterstained nucleus. **(B1–B6)** Hybridization signals corresponding with 25S rDNA. *B. stacei*-like loci (Bs' and Bs”) are pointed out by yellow arrows. **(C1–C6)** Hybridization signals corresponding with BAC clone a0019O20. Bar: 5 μm.

Supplementary Figure 2 **Confocal sections through the interphase nucleus of ***B. hybridum*** that is presented in Figure 1D. (A1–A6)** DAPI counterstained nucleus. **(B1–B6)** Hybridization signals corresponding with 25S rDNA. *B. stacei*-like loci (Bs' and Bs”) are pointed out by yellow arrows. **(C1–C6)** Hybridization signals corresponding with BAC clone a0009O09. Bar: 5 μm.

Supplementary Figure 3 **Alignment of ***B. hybridum*** and ***B. distachyon*** IGSs**. Transitions are highlighted in red and transversions in orange. InDels are visualized in blue, gaps in bolded letters.

Supplementary Figure 4 **ClustalW2 sequence alignment of the repetitive motifs Bd.B-Bh.B (A) and Bd.D-Bh.D (B) that are present in ***B. distachyon*** and ***B. hybridum*** IGSs**. Substitutions between corresponding repeat copies are highlighted in bright gray. REP motifs that have a TATA box (black frame) are highlighted in dark gray.

Supplementary Figure 5 **Confocal sections through the interphase nucleus of ***B. hybridum*** that is presented in Figures 6D1,D2. (A1–A8)** DAPI counterstained nucleus. **(B1–B8)** Hybridization signals corresponding with Bs IGS. Bar: 5 μm.

Supplementary Table 1 **Primer sequences used to amplify and sequence intergenic spacers**.

Supplementary Table 2 **Primer pairs used for the amplification of ***B. distachyon*** IGS fragments**.

Supplementary Table 3 **Blastn (megablast) analysis for ***B. hybridum*** IGS as a query**.

Supplementary Table 4 **Blastn (megablast) analysis for ***B. distachyon*** IGS as a query**.

Supplementary Table 5 **Blastn (megablast) analysis for ***B. stacei*** IGS as a query**.
