# Supplementary figures and images for: Cytomolecular Analysis of Ribosomal DNA Evolution in a Natural Allotetraploid Brachypodium hybridum and Its Putative Ancestors—Dissecting Complex Repetitive Structure of Intergenic Spacers
Source: Front Plant Sci. 2016 Oct 14;7:1499. doi: 10.3389/fpls.2016.01499 (PMC5064635; doi:10.3389/fpls.2016.01499)

Supplementary Figure 1

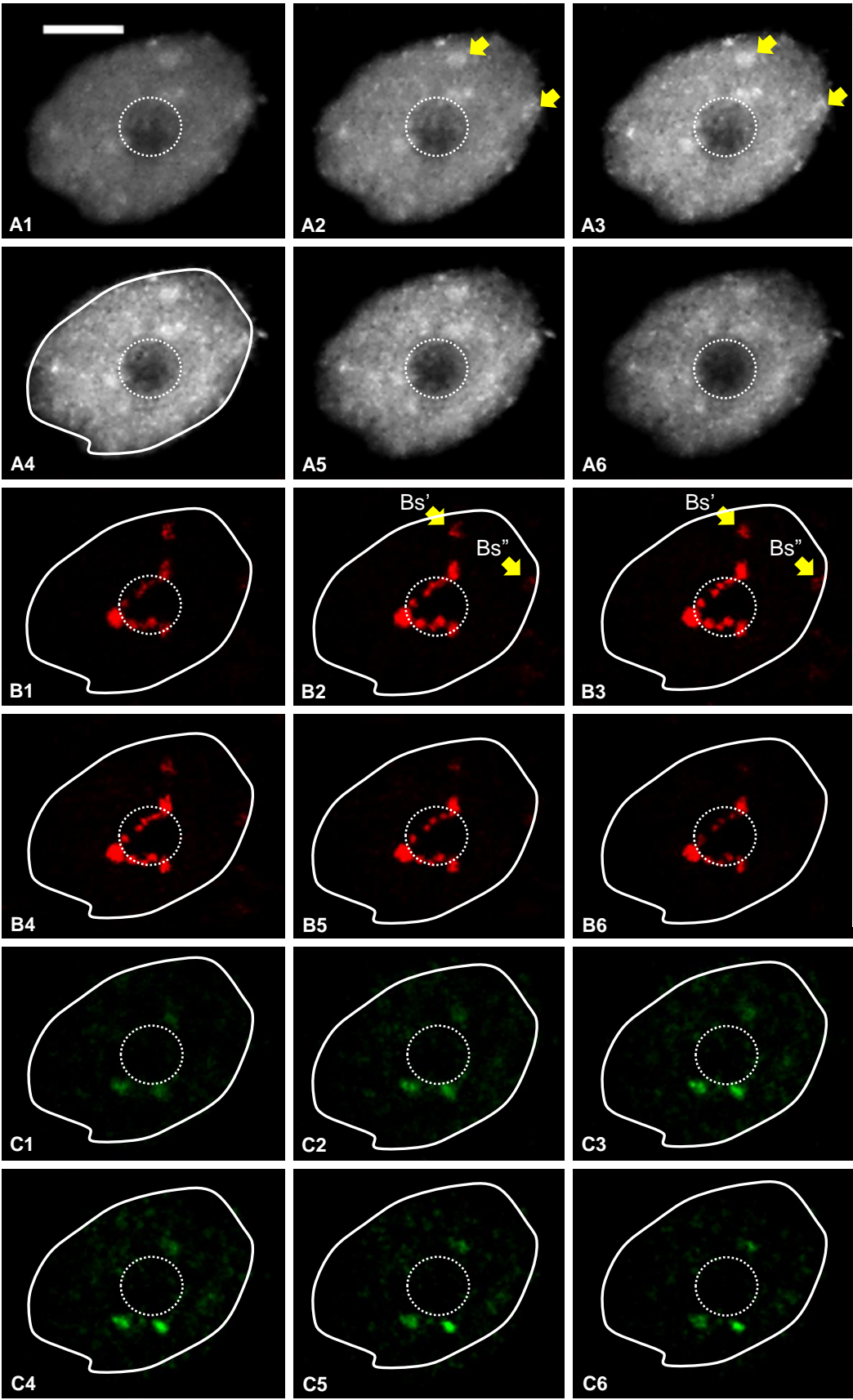

Supplement: Supplementary Figure 1 — Confocal sections through the interphase nucleus of B. hybridum that is presented in Figure 1C. (A1–A6) DAPI counterstained nucleus. (B1–B6) Hybridization signals corresponding with 25S rDNA. B. stacei-like loci (Bs' and Bs”) are pointed out by yellow arrows. (C1–C6) Hybridization signals corresponding with BAC clone a0019O20. Bar: 5 μm. [file Image1.PDF]

**Supplementary Figure 2**

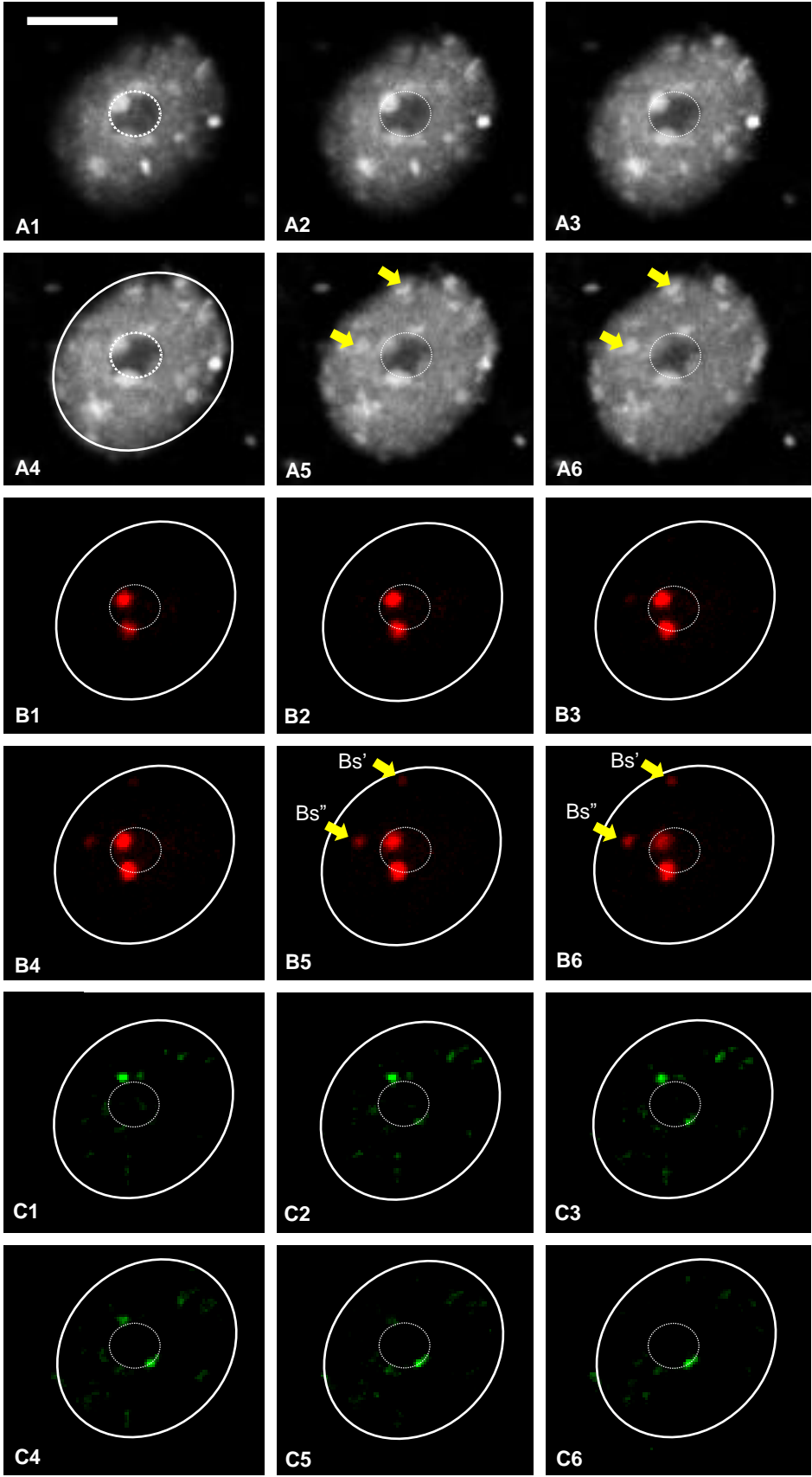

Supplement: Supplementary Figure 2 — Confocal sections through the interphase nucleus of B. hybridum that is presented in Figure 1D. (A1–A6) DAPI counterstained nucleus. (B1–B6) Hybridization signals corresponding with 25S rDNA. B. stacei-like loci (Bs' and Bs”) are pointed out by yellow arrows. (C1–C6) Hybridization signals corresponding with BAC clone a0009O09. Bar: 5 μm. [file Image2.PDF]

Supplementary Figure 5

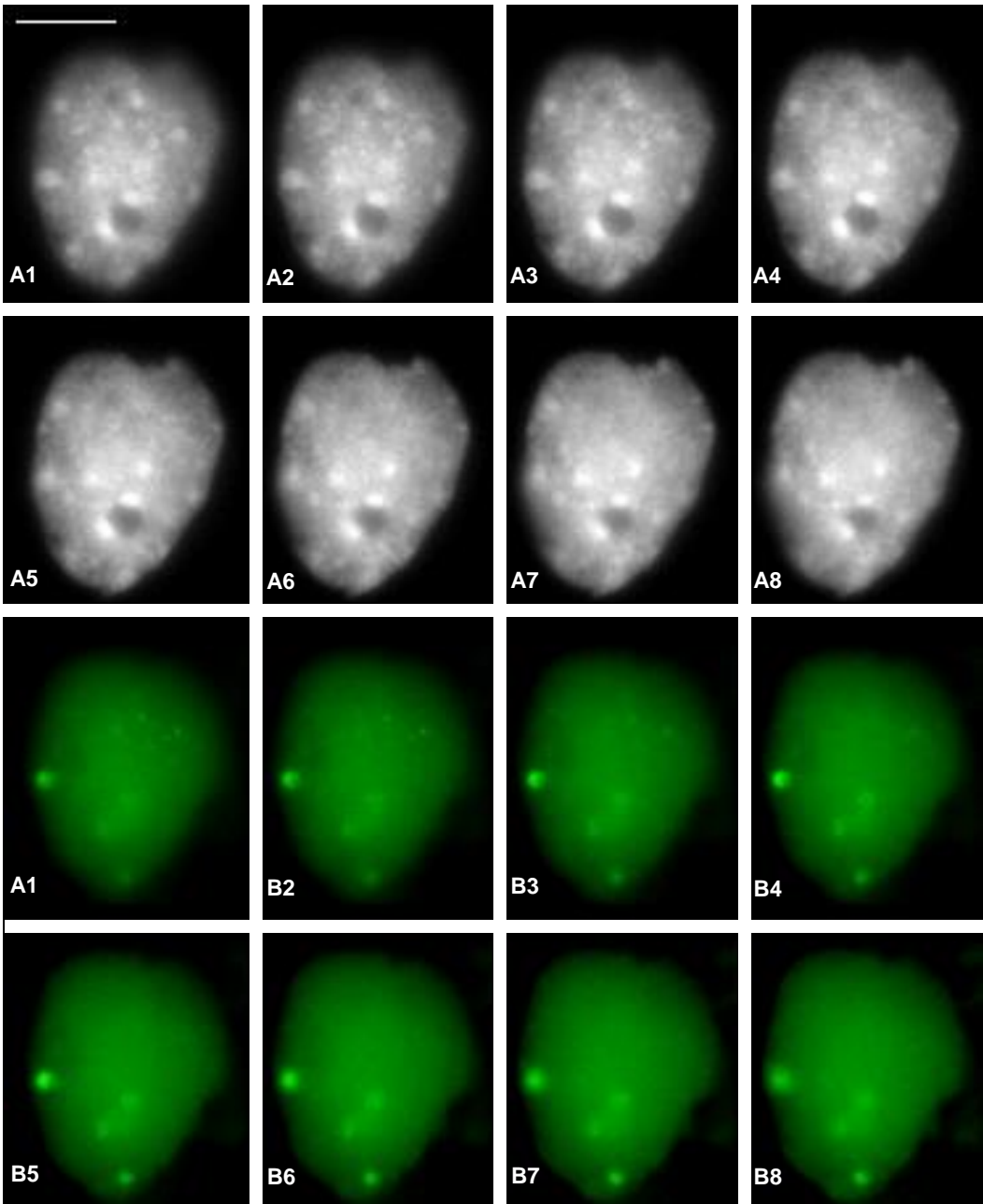

Supplement: Supplementary Figure 5 — Confocal sections through the interphase nucleus of B. hybridum that is presented in Figures 6D1,D2. (A1–A8) DAPI counterstained nucleus. (B1–B8) Hybridization signals corresponding with Bs IGS. Bar: 5 μm. [file Image5.PDF]
