# Supplementary material for: Cytomolecular Analysis of Ribosomal DNA Evolution in a Natural Allotetraploid Brachypodium hybridum and Its Putative Ancestors—Dissecting Complex Repetitive Structure of Intergenic Spacers
Source: Front Plant Sci. 2016 Oct 14;7:1499. doi: 10.3389/fpls.2016.01499 (PMC5064635; doi:10.3389/fpls.2016.01499)
Supplement: Supplementary Figure 3 — Alignment of B. hybridum and B. distachyon IGSs. Transitions are highlighted in red and transversions in orange. InDels are visualized in blue, gaps in bolded letters. [file Image3.PDF]

Supplementary Figure 3

```
Query  1    CCCTCCCCTCCCCCACCAAGGGTCGTGTCGTGCATTAGGTTCTGCCGTGCCGTCATGACA  60
            ||||||||||||||||||||||| ||||||||| ||||||||||||||||||||||||||
Sbjct  1    CCCTCCCCTCCCCCACCAAGGGTTGTGTCGTGCGTTAGGTTCTGCCGTGCCGTCATGACA  60

Query  61   TGGACGGCTTTGTTCCGCCATTGAGGAAAGCGACCAAGCGTCGCCATTGAGGAGGACGAG  120
             |||||||||||||||||||| |||||  ||||||||| |||||||||||||||||||||
Sbjct  61   CGGACGGCTTTGTTCCGCCATCGAGGAGGGCGACCAAGTGTCGCCATTGAGGAGGACGAG  120

Query  121  CAGGTTCCGCCACGAAGGAAAACGCCCGAGCGCCGCCACAGGCAATAGTTGGTGGCGCGC  180
            ||||||||||||| ||||||||||||||||||||||||||||||||||||||||||||||
Sbjct  121  CAGGTTCCGCCACCAAGGAAAACGCCCGAGCGCCGCCACAGGCAATAGTTGGTGGCGCGC  180

Query  181  ACACTCGTGGCGAAAATCGCCTTGCGCGCCCCTAAGTTGGCGGTATCGTCGCATGGCCGC  240
            |||||||||||||||| |||||||||||||||||||||||||||||||||||||||||||
Sbjct  181  ACACTCGTGGCGAAAACCGCCTTGCGCGCCCCTAAGTTGGCGGTATCGTCGCATGGCCGC  240

Query  241  CGCCCCGGACCCAGCACTAGGCCTAAATGCCATGGTCCAGGGGGGCCGTGCCCAAGGAAG  300
            ||||||||||||||||| ||||||||||||||||||||||||||| |||||||||| |||
Sbjct  241  CGCCCCGGACCCAGCACCAGGCCTAAATGCCATGGTCCAGGGGGGTCGTGCCCAAGAAAG  300

Query  301  TTGTCGGACGGCCGGATGAGTGGCATCGAGAGAAGCGTCCGCCTCGGGCGCGGAAGGCCG  360
             |||||||||||||||||||||||||||||||||||||||||||||||||||||||||||
Sbjct  301  CTGTCGGACGGCCGGATGAGTGGCATCGAGAGAAGCGTCCGCCTCGGGCGCGGAAGGCCG  360

Query  361  ATCATCGTGTTGGACGTGCGTGCACGGAAGCGGTACGGGTACCGACCTGATTCCGGCCGT  420
            |||||||||||||||||||||||||||||||||||| ||||| |||||||||||||||||
Sbjct  361  ATCATCGTGTTGGACGTGCGTGCACGGAAGCGGTACAGGTACTGACCTGATTCCGGCCGT  420

Query  421  GGCAAACGTTCTCCTCGCCGCGGCAGAGAAAATGATCGTTTTTTGACCCACGGGCAGAGG  480
            ||||||||| ||||||||||||||||||||||||||||||||||||||||||||||| ||
Sbjct  421  GGCAAACGTCCTCCTCGCCGCGGCAGAGAAAATGATCGTTTTTTGACCCACGGGCAGGGG  480

Query  481  CGACCTGCCCCTTCCTAGCGTTGTGGCAGGGAACCCGCGGGGGGCTGTCCGCCCCGGGTA  540
             |||||||||||||||||||||||||||||||||||||||||||||||||||||||||||
Sbjct  481  TGACCTGCCCCTTCCTAGCGTTGTGGCAGGGAACCCGCGGGGGGCTGTCCGCCCCGGGTA  540

Query  541  TAGTAGGGGGAGGCAGCCCCCGACACGTGCTTGTCACGGCATAGAAAATGGTCGTTTTTT  600
            |||||||||||||||||||||||||||           ||||||||||||||||||||||
Sbjct  541  TAGTAGGGGGAGGCAGCCCCCGACACG-----------GCATAGAAAATGGTCGTTTTTT  589

Query  601  GACCCGTGACTGAGGTGACCTGCCCCAACGAGGCGTTGCGGCAGGGAACCCGCGGGGGGA  660
            ||||||||||||||||||||||||||||||||||||||||||||||||||||||||||||
Sbjct  590  GACCCGTGACTGAGGTGACCTGCCCCAACGAGGCGTTGCGGCAGGGAACCCGCGGGGGGA  649

Query  661  TGTCGGCCCCGGGTATAGTAGGGTTGGAAGAAACGGAGGAACCaaaaaaaCCCACAcgcg  720
            |||||||||||||||||||||||| |||||||||||||||||||||||||||||||||||
Sbjct  650  TGTCGGCCCCGGGTATAGTAGGGTCGGAAGAAACGGAGGAACCAAAAAAACCCACACGCG  709

Query  721  cgcgcgcccgccccgcccgcgcctcgcgTCTACACGTGACTTGTGACTTGTGACTTTGGG  780
            |||||||||||||||||||||||||||||||||||||||||||||||||       ||||
Sbjct  710  CGCGCGCCCGCCCCGCCCGCGCCTCGCGTCTACACGTGACTTGTGACTT-------TGGG  762

Query  781  CTTTGGGCCTTTGGGCCTTTGGGCATATTTCGAAGCAAACCACGGTGGTTGAGCGGCGGG  840
            | ||||||||||||||||||||||||||||||||||||||||||||||||||||||||||
Sbjct  763  CCTTGGGCCTTTGGGCCTTTGGGCATATTTCGAAGCAAACCACGGTGGTTGAGCGGCGGG  822

Query  841  CACGGTCATGCCGGTCGGGTTGCCCGTTTCTCGGCCCCGCGGCATTGTTTGGGTGCTTGC  900
            ||||||||||||||||||||||||||||||||||||||||||||||||||||||||||  
Sbjct  823  CACGGTCATGCCGGTCGGGTTGCCCGTTTCTCGGCCCCGCGGCATTGTTTGGGTGCTTAT  882

Query  901  TTTGAAGGAAACCACGGGCGTTAACCCCACCGGGTACAGTTAGTCCGGTCGGGCTGGCAA  960
            |||||||||||||||||| ||||||||||||||||||||||||||||| |||||||||||
Sbjct  883  TTTGAAGGAAACCACGGGTGTTAACCCCACCGGGTACAGTTAGTCCGGCCGGGCTGGCAA  942
```

Supplementary Figure 3 (continuation)

```
Query  961   ACTCGGCAGATTTCCTGCCGCGGCAGGAATTGTCGTTGTCGCGGCAGAGGAGAGTGCCGT  1020
             |||||||||||||||||||||||||||||||||||||||||||||||||||||||| |||
Sbjct  943   ACTCGGCAGATTTCCTGCCGCGGCAGGAATTGTCGTTGTCGCGGCAGAGGAGAGTGTCGT  1002

Query  1021  CTTTTGACCCGCGGCAGCGGTCGGAAAGCGCTTCCGACCGTTGCGGCGGGGAACCCGCGG  1080
             ||||||||||||||||||||||||||||||||||||||||||| ||||||||||||||||
Sbjct  1003  CTTTTGACCCGCGGCAGCGGTCGGAAAGCGCTTCCGACCGTTGTGGCGGGGAACCCGCGG  1062

Query  1081  GGGGATGTCGGCCCCGGGTATAGTAGGGGGAGGCAGTCCCACGACGGGTCGGCTGCCGCG  1140
             ||||||||||||||||||||||||||||||||||||||||||||||||||||||||||||
Sbjct  1063  GGGGATGTCGGCCCCGGGTATAGTAGGGGGAGGCAGTCCCACGACGGGTCGGCTGCCGCG  1122

Query  1141  GCAGGAGGGTTGCCCGTTTCTCGGTCCCGCGGCATTGTTCGGGTGCTTATTTTGAAGGAA  1200
             ||||||||||||||||||||||||||||||||||||||| ||||||||||||||||||||
Sbjct  1123  GCAGGAGGGTTGCCCGTTTCTCGGTCCCGCGGCATTGTTTGGGTGCTTATTTTGAAGGAA  1182

Query  1201  ACCTCGGGTGTTAACCCCCACCGGGTACAGTTAGTCCGGTCGGGCTGGCAAACTCGGCAG  1260
             |||||||||||||||||||||||||||| |||||||||||||||||||||||||||||||
Sbjct  1183  ACCTCGGGTGTTAACCCCCACCGGGTACGGTTAGTCCGGTCGGGCTGGCAAACTCGGCAG  1242

Query  1261  ATTTCCTGCCGCGGATTCTGCCGCGGCAGGAATTGTCGTTGTCGCGGCAGAGGAAAGTGT  1320
             ||||||||||||||||||||||||||||||||||||||||||||||||||||||||||||
Sbjct  1243  ATTTCCTGCCGCGGATTCTGCCGCGGCAGGAATTGTCGTTGTCGCGGCAGAGGAAAGTGT  1302

Query  1321  CGTTTTTTGACCCGCGGCAGCGGTCGGGAAGCGCTTCCTAGCGCTGTGGCAGGGAACCCG  1380
             |||||||||||||||||||||||||| |||||||||||| ||| ||||||||||||||||
Sbjct  1303  CGTTTTTTGACCCGCGGCAGCGGTCGCGAAGCGCTTCCTGGCGTTGTGGCAGGGAACCCG  1362

Query  1381  CGGGGGGATGTCGGCCCCGGGTATAGTAGGGGGAGGCAGTCCCCCGACGGGTCGGCTGCC  1440
             ||||||||||||||||||||||||||||||||||||||||||||||||||||||||||||
Sbjct  1363  CGGGGGGATGTCGGCCCCGGGTATAGTAGGGGGAGGCAGTCCCCCGACGGGTCGGCTGCC  1422

Query  1441  GCGGCAGGAACGCGGCAGGAGTGTGGCTTTTTTCTCGGCCCGTGGCA-TTGTTCGGAAGC  1499
             ||||||||||||||||||||| ||||||||||||||||||||||||| ||||||||||||
Sbjct  1423  GCGGCAGGAACGCGGCAGGAGGGTGGCTTTTTTCTCGGCCCGTGGCATTTGTTCGGAAGC  1482

Query  1500  TAA-TTTCTGACGGAAACCACGTTGGCTGGACTCACCGAGGCAGGCCGGATCGGCGGGCG  1558
             ||| ||| ||||||||||||||||||||||||||||||||||||||||||| ||||||||
Sbjct  1483  TAATTTTTTGACGGAAACCACGTTGGCTGGACTCACCGAGGCAGGCCGGATGGGCGGGCG  1542

Query  1559  CGGCAGAAATCCTGCCTCGTCCGCAGGGTTGCCGTTTTTCGTGCCCGTAACATCGTCCGG  1618
             ||||||||||||||||||||||| ||||||||||||||||| |||||||||||||| |||
Sbjct  1543  CGGCAGAAATCCTGCCTCGTCCGTAGGGTTGCCGTTTTTCGCGCCCGTAACATCGTTCGG  1602

Query  1619  AGGCTTATTTGAAGGAAACCACGGGTGTTTCGACCCCCAGGTATAAAGCA----------  1668
             |||||| |||||||||||||||||||||||||||||||||||||||||||
Sbjct  1603  AGGCTTGTTTGAAGGAAACCACGGGTGTTTCGACCCCCAGGTATAAAGCAGCGGTTGCCT  1662

Query  1669  --GCGGTCCTGCCGTGGCAGGAAAACGGCCCGTCCTCTTGCGCCCGTGT-----------  1715
               |||||||||||||||||||| |||||||||| |||||||||||||||
Sbjct  1663  CGGCGGTCCTGCCGTGGCAGGAGAACGGCCCGTTCTCTTGCGCCCGTGTCATGTTGCCTC  1722

Query  1716  ------------------CATCGTAACTCCCGCGCGCTGTGGACCTTCGGTCGCCGTATT  1757
                               ||||||||||||||||||||||||||||||||||||||||||
Sbjct  1723  GAAAATCCTTTGCGCTGCCATCGTAACTCCCGCGCGCTGTGGACCTTCGGTCGCCGTATT  1782

Query  1758  GAGTAGACCCGCCGTGCTCTGCGCGGTGTGATGCTTGTACGGTCTGAACGTCGTGGCTAC  1817
             ||||||||||||||||||||||||||||||||||||||||||| ||||||||||||||||
Sbjct  1783  GAGTAGACCCGCCGTGCTCTGCGCGGTGTGATGCTTGTACGGTTTGAACGTCGTGGCTAC  1842

Query  1818  GCTAGCGCATGAGTTGTCTTGGACCAGTGTCTGCCGGCAGATCCCCCGCCGTTGTGCGGC  1877
             ||||||||||||||||||||||||||||||||||||||||||||||||||||||||||||
Sbjct  1843  GCTAGCGCATGAGTTGTCTTGGACCAGTGTCTGCCGGCAGATCCCCCGCCGTTGTGCGGC  1902

Query  1878  CGACTACCGGCGCCTGTGTCCTATCAACGTTGCATTGGACGGCTCTTACTTTTGCCGCCT  1937
             ||||||||||||||||||||||||||||||||||||||||||||||||| ||||||||||
Sbjct  1903  CGACTACCGGCGCCTGTGTCCTATCAACGTTGCATTGGACGGCTCTTAC-TTTGCCGCCT  1961
```

Supplementary Figure 3 (continuation)

```
Query  1938   TACCGGAAAGTTCTTGCATGATGGTCCCCGACCAACGGGAATTGGTGCGTCGTAGAGAGT  1997
              ||||||||||||||||||||||||||||||||||||||||||||||||||||||||||||
Sbjct  1962   TACCGGAAAGTTCTTGCATGATGGTCCCCGACCAACGGGAATTGGTGCGTCGTAGAGAGT  2021

Query  1998   AGCCTCGCGGTGGATGCCTGTTGGCGTTCCACTGCGGCCATATTGCGTTGGCGGCGTTGC  2057
              ||||||||||||||||||||||||||||||||||||||||||||||||||||||||||||
Sbjct  2022   AGCCTCGCGGTGGATGCCTGTTGGCGTTCCACTGCGGCCATATTGCGTTGGCGGCGTTGC  2081

Query  2058   CTCGTGGTGCCATCACGCTACGTGTTTGGCCCTACCAAGGACACCTCGCTCCCGCTCTTG  2117
              ||||||||||||||||||||||||||||||||||||||||||||||||||||||||||||
Sbjct  2082   CTCGTGGTGCCATCACGCTACGTGTTTGGCCCTACCAAGGACACCTCGCTCCCGCTCTTG  2141

Query  2118   GTCTCGGATGTCGCTCATTGTAGAGGCTCGTGGCCCTTTGGCGTCGCGTCCCTTTCTGAA  2177
              |||| |||||||||||||||||||||||||||||||||||||||||||||||||||||||
Sbjct  2142   GTCTTGGATGTCGCTCATTGTAGAGGCTCGTGGCCCTTTGGCGTCGCGTCCCTTTCTGAA  2201

Query  2178   GCTCACCCAAAGGACGACAGCCGCTCCGtttttttGCCGTTGCCATGGCATGCAAGTGGC  2237
              |||||| ||||||||||||||||||||| ||||| |||||||||||||||||||||||||
Sbjct  2202   GCTCACTCAAAGGACGACAGCCGCTCCG-TTTTTCGCCGTTGCCATGGCATGCAAGTGGC  2260

Query  2238   TGGCACGCGCGACTCTCGGTGACGGCTTGTCGACTAGGACGTGCT  2282
              ||||||| |||||||||||||||||||||||||||||||||||||
Sbjct  2261   TGGCACGTGCGACTCTCGGTGACGGCTTGTCGACTAGGACGTGCT  2305
```
