# Supplementary material for: Cytomolecular Analysis of Ribosomal DNA Evolution in a Natural Allotetraploid Brachypodium hybridum and Its Putative Ancestors—Dissecting Complex Repetitive Structure of Intergenic Spacers
Source: Front Plant Sci. 2016 Oct 14;7:1499. doi: 10.3389/fpls.2016.01499 (PMC5064635; doi:10.3389/fpls.2016.01499)
Supplement: Supplementary Figure 4 — ClustalW2 sequence alignment of the repetitive motifs Bd.B-Bh.B (A) and Bd.D-Bh.D (B) that are present in B. distachyon and B. hybridum IGSs. Substitutions between corresponding repeat copies are highlighted in bright gray. REP motifs that have a TATA box (black frame) are highlighted in dark gray. [file Image4.PDF]

Supplementary Figure 4

A

```
Bd.B_1        CGGCAGAGAAAATGATCGTTTTTTGACCCACGGGCAGAGGCGACCTGCCCCTTCCTAGCG 60
Bd.B_2        CGGCATAGAAAATGGTCGTTTTTTGACCCGTG-ACTGAGGTGACCTGCCCCAACGAGGCG 59
Bh.B_1        CGGCAGAGAAAATGATCGTTTTTTGACCCACGGGCAGGGGTGACCTGCCCCTTCCTAGCG 60
Bh.B_2        CGGCATAGAAAATGGTCGTTTTTTGACCCGTG-ACTGAGGTGACCTGCCCCAACGAGGCG 59
              ***** ********.**************. * .*:*.** **********::* :.***

Bd.B_1        TTGTGGCAGGGAACCCGCGGGGGGCTGTCCGCCCCGGGTATAGTAGGGGGAGGCAGCCCC 120
Bd.B_2        TTGCGGCAGGGAACCCGCGGGGGGATGTCGGCCCCGGGTATAGTAGGG------------ 107
Bh.B_1        TTGTGGCAGGGAACCCGCGGGGGGCTGTCCGCCCCGGGTATAGTAGGGGGAGGCAGCCCC 120
Bh.B_2        TTGCGGCAGGGAACCCGCGGGGGGATGTCGGCCCCGGGTATAGTAGGG------------ 107
              *** ********************.**** ******************

Bd.B_1        CGACA 125
Bd.B_2        -----
Bh.B_1        CGACA 125
Bh.B_2        -----
```

B

```
Bd.D_I        GGGTTGCCCGTTTCTCGGCCCCGCGGCATTGTTTGGGTGCTTGCTTTGAAGGAAACCACG 60
Bh.D_I        GGGTTGCCCGTTTCTCGGCCCCGCGGCATTGTTTGGGTGCTTATTTTGAAGGAAACCACG 60
Bd.D_II       GGGTTGCCCGTTTCTCGGTCCCGCGGCATTGTTCGGGTGCTTATTTTGAAGGAAACCTCG 60
Bh.D_II       GGGTTGCCCGTTTCTCGGTCCCGCGGCATTGTTTGGGTGCTTATTTTGAAGGAAACCTCG 60
              ****************** ************** ********. *************:**

Bd.D_I        GGCGTTAACCCC-ACCGGGTACAGTTAGTCCGGTCGGGCTGGCAAACTCGGCAGATTTCC 119
Bh.D_I        GGTGTTAACCCC-ACCGGGTACAGTTAGTCCGGCCGGGCTGGCAAACTCGGCAGATTTCC 119
Bd.D_II       GGTGTTAACCCCCACCGGGTACAGTTAGTCCGGTCGGGCTGGCAAACTCGGCAGATTTCC 120
Bh.D_II       GGTGTTAACCCCCACCGGGTACGGTTAGTCCGGTCGGGCTGGCAAACTCGGCAGATTTCC 120
              ** ********* *********.********** **************************

Bd.D_I        TGCCGCG------------GCAGGAATTGTCGTTGTCGCGGCAGAGGAGAGTGCCGTCTT 167
Bh.D_I        TGCCGCG------------GCAGGAATTGTCGTTGTCGCGGCAGAGGAGAGTGTCGTCTT 167
Bd.D_II       TGCCGCGGATTCTGCCGCGGCAGGAATTGTCGTTGTCGCGGCAGAGGAAAGTGTCGTTTT 180
Bh.D_II       TGCCGCGGATTCTGCCGCGGCAGGAATTGTCGTTGTCGCGGCAGAGGAAAGTGTCGTTTT 180
              *******            *****************************.**** *** **

Bd.D_I        TTGACCCGCGGCAGCGGTCGGAAAGCGCTTCCGACCGTTGCGGCGGGGAACCCGCGGGGG 227
Bh.D_I        TTGACCCGCGGCAGCGGTCGGAAAGCGCTTCCGACCGTTGTGGCGGGGAACCCGCGGGGG 227
Bd.D_II       TTGACCCGCGGCAGCGGTCGGGAAGCGCTTCCTAGCGCTGTGGCAGGGAACCCGCGGGGG 240
Bh.D_II       TTGACCCGCGGCAGCGGTCGCGAAGCGCTTCCTGGCGTTGTGGCAGGGAACCCGCGGGGG 240
              ******************** .********** . ** ** ***.***************

Bd.D_I        GATGTCGGCCCCGGGTATAGTAGGGGGAGGCAGTCCCACGACGGGTCGGCTGCCGCGGCA 287
Bh.D_I        GATGTCGGCCCCGGGTATAGTAGGGGGAGGCAGTCCCACGACGGGTCGGCTGCCGCGGCA 287
Bd.D_II       GATGTCGGCCCCGGGTATAGTAGGGGGAGGCAGTCCCCCGACGGGTCGGCTGCCGCGGCA 300
Bh.D_II       GATGTCGGCCCCGGGTATAGTAGGGGGAGGCAGTCCCCCGACGGGTCGGCTGCCGCGGCA 300
              *************************************.**********************

Bd.D_I        GGA 290
Bh.D_I        GGA 290
Bd.D_II       GGA 303
Bh.D_II       GGA 303
              ***
```
