# Supplementary material for: Cytomolecular Analysis of Ribosomal DNA Evolution in a Natural Allotetraploid Brachypodium hybridum and Its Putative Ancestors—Dissecting Complex Repetitive Structure of Intergenic Spacers
Source: Front Plant Sci. 2016 Oct 14;7:1499. doi: 10.3389/fpls.2016.01499 (PMC5064635; doi:10.3389/fpls.2016.01499)
Supplement: Supplementary Table 1 — Primer sequences used to amplify and sequence intergenic spacers. [file Table1.PDF]

Supplementary Table 1. Primer sequences used to amplify and sequence intergenic spacers.

	Primer name	Sequence
**IGS Amplification**	25S forward primer	5’-TTGCTGCCACGATCCACTGAG-3’
	18S reverse primer	5’-CTACTGGCAGGATCAACCAGG-3’
***B. hybridum* IGS sequencing**	M13for	5’-GTAAAACGACGGCCAGT-3’
	For1	5’-CGCGGCAGAGAAAATGAT-3’
	For2	5’-ATAGTAGGGGGAGGCAGTCC-3’
	For3	5’-CCTGCCGCGGATT-3’
	M13rev	5’-CAGGAAACAGCTATGAC-3’
