# Supplementary material for: Cytomolecular Analysis of Ribosomal DNA Evolution in a Natural Allotetraploid Brachypodium hybridum and Its Putative Ancestors—Dissecting Complex Repetitive Structure of Intergenic Spacers
Source: Front Plant Sci. 2016 Oct 14;7:1499. doi: 10.3389/fpls.2016.01499 (PMC5064635; doi:10.3389/fpls.2016.01499)
Supplement: Supplementary Table 2 — Primer pairs used for the amplification of B. distachyon IGS fragments. [file Table2.PDF]

**Supplementary Table 2.** Primer pairs used for the amplification of *B. distachyon* IGS fragments.

| Forward Primer | Reverse Primer |
|---|---|
| **F1:** 5’-TTGCTGCCACGATCCACTGAG-3’ | **R1:** 5’-CTCTGCCCGTGGGTCAAAA-3’ |
| **F2:** 5’-TGAGTGGCATCGAGAGAAGC-3’ | **R2:** 5’-GACAACGACAATTCCTGCCG-3’ |
| **F3:** 5’-ATGCCGGTCGGGTTGCC-3’ | **R3:** 5’-AGCCAACGTGGTTTCCGTC-3’ |
| **F4:** 5’-CGGCAGGAGTGTGGCTTTTT-3’ | **R4:** 5’-AGGCTACTCTCTACGACGCA-3’ |
| **F5:** 5’-TTGCATTGGACGGCTCTTACT-3’ | **R5:** 5’-CTACTGGCAGGATCAACCAGG-3’ |
