# Supplementary material for: Cytomolecular Analysis of Ribosomal DNA Evolution in a Natural Allotetraploid Brachypodium hybridum and Its Putative Ancestors—Dissecting Complex Repetitive Structure of Intergenic Spacers
Source: Front Plant Sci. 2016 Oct 14;7:1499. doi: 10.3389/fpls.2016.01499 (PMC5064635; doi:10.3389/fpls.2016.01499)
Supplement: Supplementary Table 3 — Blastn (megablast) analysis for B. hybridum IGS as a query. [file Table3.PDF]

Supplementary Table 3. Blastn (megablast) analysis for *B. hybridum* IGS as a query.

Sequence name (GeneBank)*	Species	Query cover	Identity	E value
Brachypodium sp. MP-2011a isolate Bdis403 external transcribed spacer, partial sequence (JN187595.1)	*Brachypodium hybridum* (Spain)	19%	100%	0.0
Brachypodium sp. MP-2011a isolate Bdis117 external transcribed spacer, partial sequence (JN187590.1)	*Brachypodium hybridum* (Afghanistan)	19%	99%	0.0
Brachypodium sp. MP-2011a isolate Bdis110 external transcribed spacer, partial sequence (JN187587.1)	*Brachypodium hybridum* (France)	19%	99%	0.0
Brachypodium distachyon isolate Bdis306 external transcribed spacer, partial sequence (JN187591.1)	*Brachypodium distachyon* (Spain)	19%	99%	0.0
Brachypodium distachyon isolate Bdis400 external transcribed spacer, partial sequence (JN187592.1)	*Brachypodium distachyon* (Spain)	19%	99%	0.0

* Only the first five results are presented in the table.
