# Supplementary material for: Cytomolecular Analysis of Ribosomal DNA Evolution in a Natural Allotetraploid Brachypodium hybridum and Its Putative Ancestors—Dissecting Complex Repetitive Structure of Intergenic Spacers
Source: Front Plant Sci. 2016 Oct 14;7:1499. doi: 10.3389/fpls.2016.01499 (PMC5064635; doi:10.3389/fpls.2016.01499)
Supplement: Supplementary Table 5 — Blastn (megablast) analysis for B. stacei IGS as a query. [file Table5.PDF]

Supplementary Table 5. Blastn (megablast) analysis for *B. stacei* IGS as a query.

Sequence name (GeneBank)*	Species	Query cover	Identity	E value
Brachypodium sp. MP-2011b isolate Bdis114 external transcribed spacer, partial sequence (JN187589.1)	*Brachypodium stacei* (Spain)	19%	100%	0.0
Brachypodium mexicanum isolate Bmex347 external transcribed spacer, partial sequence (JN187596.1)	*Brachypodium mexicanum* (Mexico)	19%	91%	7e-175
Brachypodium rupestre isolate BrupPC2393 external transcribed spacer, partial sequence (JN187600.1)	*Brachypodium rupestre* (Spain)	19%	90%	7e-170
Brachypodium retusum isolate Bret1 external transcribed spacer, partial sequence (JN187599.1)	*Brachypodium retusum* (Spain)	19%	90%	3e-168
Brachypodium phoenicoides isolate Bpho39 external transcribed spacer, partial sequence (JN187597.1)	*Brachypodium phoenicoides* (France)	19%	90%	3e-168

* Only the first five results are presented in the table.
